# A monolithically integrated near-infrared imager with crystallization- and oxidation-modulated tin-lead perovskites

**DOI:** 10.1038/s41377-025-01987-8

**Published:** 2025-09-04

**Authors:** Zhichun Yang, Jingjing Liu, Haotian Bao, Zonghao Liu, Zaiwei Wang, Xiangdong Li, Zhihao Chen, Guofeng Zhang, Ruiyun Chen, Jianyong Hu, Shuangping Han, Wei Chen, Chengbing Qin, Liantuan Xiao, Suotang Jia

**Affiliations:** 1 State Key Laboratory of Quantum Optics Technologies and Devices, Institute of Laser Spectroscopy, Shanxi University, 030006 Taiyuan, China; 2 Collaborative Innovation Center of Extreme Optics, Shanxi University, 030006 Taiyuan, China; 3 Wuhan National Laboratory for Optoelectronics, Huazhong University of Science and Technology, 430074 Wuhan, China; 4 Faculty of Materials Science and Energy Engineering, Shenzhen University of Advanced Technology, 518107 Shenzhen, China; 5 College of Physics and Optoelectronic Engineering, Taiyuan University of Technology, 030600 Taiyuan, China

**Keywords:** Optoelectronic devices and components, Imaging and sensing

## Abstract

The fast crystallization and facile oxidation of Sn^2+^ of tin-lead (Sn-Pb) perovskites are the biggest challenges for their applications in high-performance near-infrared (NIR) photodetectors and imagers. Here, we introduce a multifunctional diphenyl sulfoxide (DPSO) molecule into perovskite precursor ink to response these issues by revealing its strong binding interactions with the precursor species. The regulated perovskite film exhibits a dense morphology, reduced defect density and prolonged carrier diffusion length. The manufactured self-powered photodetector realizes a spectral response of 300-1100 nm, dark current density of 4.7 × 10^−8^ mA cm^−2^, peak responsivity of 0.49 A W^−1^ and specific detectivity of 1.20 × 10^12^ Jones in NIR region (780–1100 nm), –3 dB bandwidth of 11.4 MHz, linear dynamic range of 174 dB, and ultrafast rise/fall time of 14.2/17.1 ns, respectively. We demonstrate a 64 × 64 NIR imager with an impressive spatial resolution of 1.32 lp mm^−1^ by monolithically integrating the photodetector with a commercial thin-film transistor readout circuit.

## Abstract (web summary)

A monolithically integrated near-infrared imager is achieved with the crystallization- and oxidation-modulated tin-lead perovskites.

## Graphical abstract

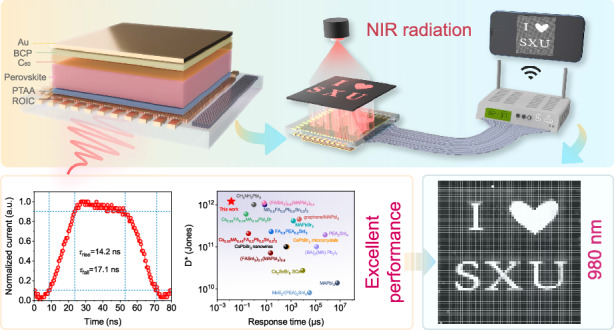

## Introduction

Near-infrared (NIR) imagers have demonstrated widespread applications in both military and civilian fields, including security monitoring^1^, space exploration^2^, night vision^3^, optical communications^4–6^, automatic driving^7,8^, and bioimaging^9,10^. They are generally stacked by the photodiodes and complementary metal-oxide-semiconductor (CMOS) or thin-film transistor (TFT) readout integrated circuitry (ROIC)^11^. Thus, the processing compatibility between the photodiodes and ROIC backplanes is highly required. Despite the traditional inorganic semiconductors, such as silicon (Si), germanium (Ge), and indium gallium arsenide (InGaAs), are the current choices for NIR photodetectors, their high-temperature and vacuum processes are great challenges for the manufacturing cost and flexible compatibility. Besides, the heterogeneous integration between Ge or InGaAs semiconductors and the silicon-based ROIC is also demanding. Alternatively, solution-processable technologies, such as lead sulfide (PbS) colloidal quantum dots^12,13^, organic materials^3,14^, and the emerging metal halide perovskite semiconductors^15–17^, have exhibited competitive advantages, especially in terms of their direct monolithic integration with the well-established ROIC technology and flexible applications in the intelligent consumer electronics.

Tin–lead (Sn–Pb) perovskites have been considered as one of the most promising candidates for high-performance NIR photodetectors on account of their remarkable properties of narrow bandgap^18^, broad spectrum response^19^, high optical absorption coefficient and carrier mobility^20^, low exciton binding energy^21^, long carrier diffusion length^16^, and ease of fabrication. However, the device performance is still dramatically limited by the inferior quality of perovskite films due to the rapid crystallization and easy oxidation of Sn^2+^ of Sn–Pb perovskites. On the one hand, the high Lewis acidity of Sn^2+^ results in the rapid crystallization of Sn-containing perovskites, which is responsible for the formation of undesired cracks and pinholes in film morphology and poor crystal quality^21,22^. On the other hand, the high reactivity of the electrons in the 5*s* orbital of Sn^2+^ contributes to its facile oxidation^23^, which will induce numerous Sn vacancy defects. Besides, the ultralow dark current, high detectivity, and fast response for the photodetectors, as well as their monolithic integration with COMS or TFT readout circuit, are still an impending step to promote the practical scenario application of NIR imagers based on Sn–Pb perovskites.

In this article, we demonstrate an NIR imager by monolithically integrating the developed Sn–Pb perovskite photodetectors with a commercial silicon-based TFT readout circuit. To regulate the crystallization and oxidation of Sn^2+^ of Sn–Pb perovskite films, a multifunctional molecule of diphenyl sulfoxide (DPSO) has been introduced into the configured perovskite precursor ink. The retarded crystallization and suppressed oxidation of Sn^2+^ of Sn–Pb perovskites have been successfully achieved based on the Lewis acid-base interaction. The DPSO-modulated perovskite film exhibits a dense surface morphology and reduced roughness, which is beneficial for the preferable interface contact between the perovskite film and the electron transport layer (ETL). Impressively, the defect density in the target perovskite film has been reduced owing to the decrease in the undercoordinated metal ions. Consequently, the fabricated Sn–Pb perovskite NIR photodetectors operated at zero bias achieved a dark current density of 4.7 × 10^−8^ mA cm^-2^, peak responsivity of 0.49 A W^−1^ and detectivity of 1.20 × 10^12^ Jones in NIR region (780–1100 nm), –3 dB bandwidth of 11.4 MHz, linear dynamic range (LDR) of 174 dB, and ultrafast response speed (14.2 ns for rise time and 17.1 ns for fall time). Furthermore, we fabricate 5 × 5 photodetector arrays, and they demonstrate satisfactory imaging ability under NIR radiation. Finally, we demonstrate a 64 × 64 NIR imager with an impressive spatial resolution of 1.32 lp mm^−1^ by monolithically integrating the photodetector with a commercial TFT readout circuit.

## Results

### Density functional theory (DFT) analysis of molecular interactions

**Fig. 1:**
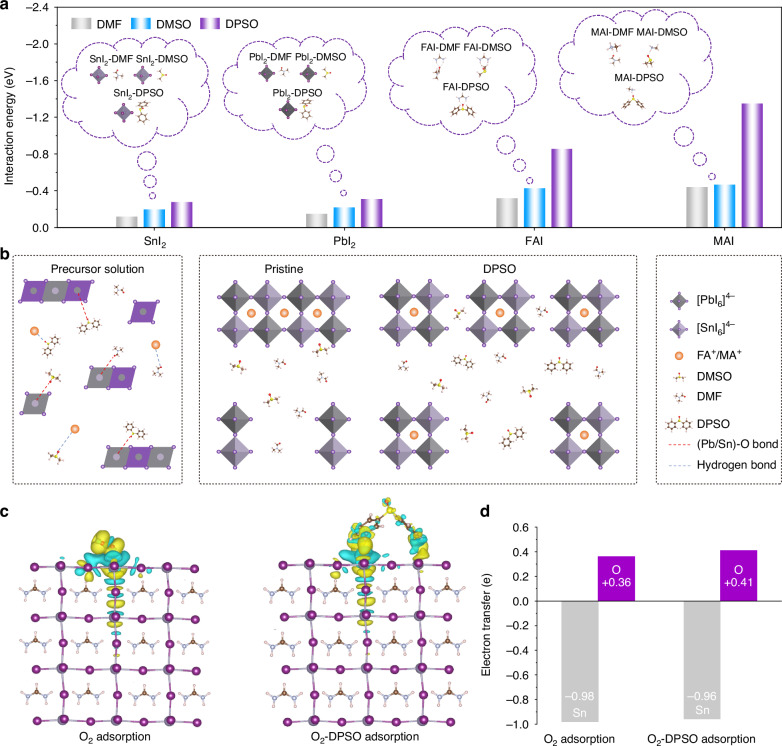
DFT analysis of the crystallization of Sn–Pb perovskites and the oxidation of Sn^2+^. **a** Interaction energy histogram between the precursor species and the solvent/introduced DPSO molecules. **b** Schematic diagram of intermolecular bonding in the precursor inks. **c** Electron density distribution on the surface of the typical FASnI_3_ perovskites after O_2_ and O_2_-DPSO adsorption. **d** Electron transfer of O atoms in O_2_ and Sn^2+^ in FASnI_3_ perovskites under O_2_ and O_2_-DPSO adsorption

Lewis base molecule of DPSO was introduced into the prepared perovskite precursor ink, in view of its strong Lewis basicity^24^ and nonvolatile property at low processing temperatures (<150 °C)^25^. To evaluate the molecular interactions between the used solvents or DPSO molecules and the precursor species in the designed Sn–Pb perovskite solution with a typical composition of FA_0.7_MA_0.3_Pb_0.5_Sn_0.5_I_3_, DFT analysis was carried out. As shown in Fig. 1a, the interaction energies of various phases are demonstrated. It can be found that DPSO exhibits stronger interactions with both SnI_2_ and PbI_2_ than the counterparts of the involved solvent molecules of dimethylformamide (DMF) and dimethyl sulfoxide (DMSO), potentially raising a higher energy barrier for the crystallization of Sn–Pb perovskites. The strong molecular interactions are beneficial for reducing the undercoordinated Sn^2+^ and Pb^2+^ defects. Besides, the DPSO molecule demonstrates the highest binding energy with both FAI and MAI molecules compared to that of DMF and DMSO, which is proposed to be derived from the formation of hydrogen bonds as illustrated in Fig. 1b.

It is widely acknowledged that the oxidation of Sn^2+^ is generally induced by the adsorption of oxygen molecules (O_2_) on the surface of Sn-containing perovskites^20,26^. For this purpose, the electron density distributions of FASnI_3_ perovskites under O_2_ and O_2_-DPSO adsorption were typically performed to assess the suppression of Sn^2+^ oxidation (Fig. 1c). It has been verified that the charge density has a prominent impact on the charge transfer^27^. The calculated electron transfer results of FASnI_3_ perovskites under O_2_ and O_2_-DPSO adsorption are exhibited in Fig. 1d. Note that the surface unsaturated Sn^2+^ in pristine perovskite tends to lose 0.98e with the adsorption of O_2_. In comparison, it is decreased to 0.96e with the adsorption of O_2_-DPSO. The reduced charge transfer implies a higher energy barrier for Sn^2+^ oxidation^27,28^. In addition, DPSO has a great potential to provide an electronic environment on account of its electron-pair donor property, which is unfavorable for Sn^2+^ oxidation.

### Modulation of crystallization and oxidation of Sn–Pb perovskites

**Fig. 2:**
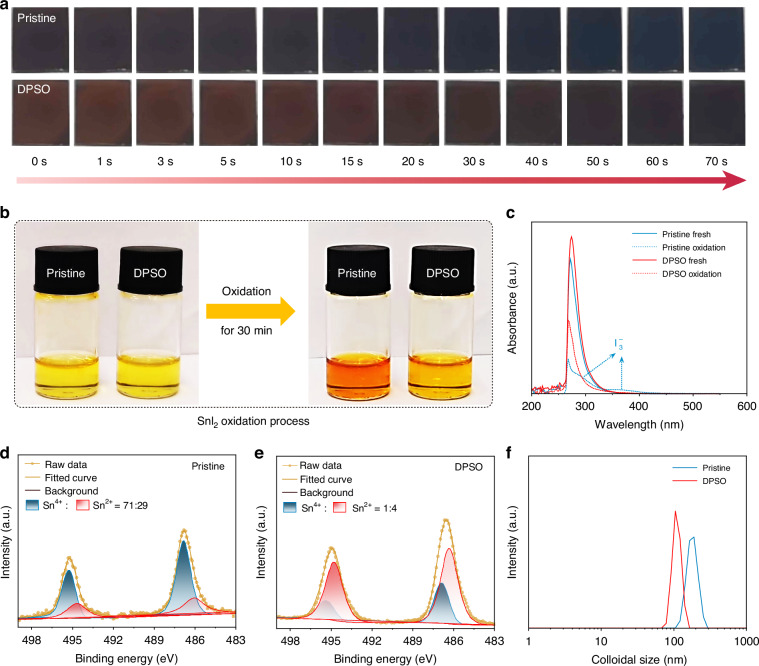
Characterization of crystallization and oxidation of Sn–Pb perovskites. **a** Natural crystallization process of Sn–Pb perovskite intermediate phases without (Pristine) and with DPSO modulation (DPSO). **b** Oxidation of SnI_2_ solution (dissolved in DMF) without and with DPSO. **c** In-situ UV–vis absorption spectra of SnI_2_ solutions at a concentration of 1 mM. XPS spectra of Sn 3*d* of **d** pristine, and **e** DPSO-modulated Sn–Pb perovskite films. **f** DLS of perovskite precursor solutions

To investigate the impact of the DPSO molecule on the crystallization of Sn–Pb perovskites, the natural crystallization processes of their intermediate state films were recorded. As shown in Fig. 2a, Supplementary videos 1 and 2, it is evident that the color change from reddish brown to black is slowed down for the DPSO-modulated film compared with the pristine sample, indicating a retarded crystallization of Sn–Pb perovskites. The delayed crystallization is ascribed to the elevated energy barrier, which is consistent with the above DFT analysis.

In terms of the suppressed oxidation of Sn^2+^, the oxidation of SnI_2_ dissolved in DMF and DMF/DPSO was comparatively carried out. As demonstrated in Fig. 2b and Supplementary video 3, the color change from pale yellow to distinct brown for the solution with DPSO (denoted as DPSO) was less noticeable compared to the ink without DPSO (named as Pristine), after they were exposed to O_2_ and aging for 30 min. The brown color generally implies the oxidation of Sn^2+^. This result can be attributed to the enhanced energy barrier for the adsorption of O_2_ to Sn^2+^, which has been analyzed by the DFT calculation. Moreover, it was also confirmed by the in-situ ultraviolet-visible (UV–vis) absorption spectra of SnI_2_ solutions (1 mM). As exhibited in Figs. 2c and S1, it is obvious that two absorption peaks located at the wavelength of 293 and 365 nm were significantly detected after the pristine SnI_2_ solution was exposed in ambient air for over 50 min, which could be ascribed to the generation of I_3_^–^. In comparison, the peaks are inapparent in the DPSO-modulated SnI_2_ solution even after 100 min. Generally, the oxidation degree of Sn^2+^ can be evaluated by the oxidation of I^–^ to ${{\rm{I}}}_{3}^{-}$, due to the precedent oxidation of Sn^2+^ than I^–^ $\left({{\rm{Sn}}}^{2+}\rightleftharpoons {{\rm{Sn}}}^{4+}+{2{\rm{e}}}^{-},{3{\rm{I}}}^{-}\rightleftharpoons {{\rm{I}}}_{3}^{-}+{2{\rm{e}}}^{-}\right)$^29,30^. Furthermore, the content of Sn^2+^ in the final Sn–Pb perovskite films was further analyzed by X-ray photoelectron spectroscopy (XPS). As shown in Fig. 2d and e, the content of Sn^4+^ is obviously decreased for the DPSO-modulated perovskite film compared to the pristine sample, indicating the effect of suppressing Sn^2+^ oxidation by DPSO.

To reveal the underlying mechanisms for the modulated crystallization of Sn–Pb perovskites and the suppressed oxidation of Sn^2+^, dynamic light scattering (DLS), XPS, and Fourier transform infrared (FTIR) were subsequently carried out. Firstly, the impact of the DPSO molecule on the colloidal property of perovskite precursor inks was investigated by DLS. As illustrated in Fig. 2f, the DPSO-modulated solution exhibits a narrower and smaller colloid size distribution than the pristine sample, which is conducive to enhancing the energy barrier for nucleation and retarding the crystallization of Sn–Pb perovskites^31^. The decreased cluster size in DPSO-modulated precursor solution is proposed to be related to the increased solubility^32^, which originates from the strong interactions between the DPSO molecule and precursor species. Secondly, the binding energy shifts of Pb 4*f* and I 3*d* (Fig. S2) in XPS results can further confirm the interactions between the DPSO molecule and perovskites. In addition, their interactions can also be verified by FTIR. As shown in Fig. S3a, it can be found that the S = O stretching vibration peak of the bare DPSO molecule at 1037 cm^−1^ shifts to 940 cm^−1^ in SnI_2_–DPSO powders, which is ascribed to the coordination between DPSO and Sn^2+^. In addition, it shifts to 954 cm^−1^ for PbI_2_–DPSO complex, indicating the interaction between Pb^2+^ and DPSO^25^. With respect to the interactions between DPSO and organic amine salts, including FAI and MAI, FTIR was performed for FAI−DPSO and MAI−DPSO powders. The N−H stretching vibration peaks in both FAI−DPSO and MAI−DPSO exhibit significant shifts towards the lower wavenumber compared to that in bare FAI and MAI, respectively (Fig. S3b), revealing the formation of hydrogen bonds between DPSO and the organic cations^33^.

### Quality of Sn–Pb perovskite films

**Fig. 3:**
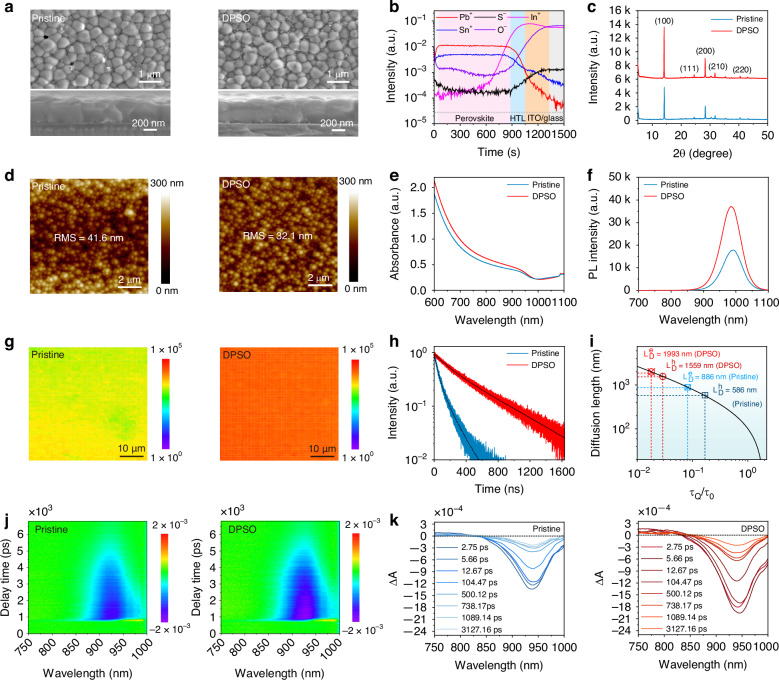
Properties of Sn–Pb perovskite films. **a** Top-view and cross-sectional SEM images of the pristine and DPSO-modulated perovskite films. **b** ToF-SIMS of the DPSO-modulated perovskite film deposited on PTAA/ITO substrate. **c** XRD, **d** AFM images, **e** UV–vis–NIR spectra, **f** PL spectra, **g** PL mapping, **h** TRPL spectra, and **i** carrier diffusion length of the deposited perovskite films. **j** 2D pseudocolor map of the perovskite films. **k** TAS curves at different probe delays for the pristine and DPSO-modulated films

To investigate the impact of introducing the DPSO molecule on the properties of Sn–Pb perovskite films, scanning electron microscopy (SEM) was first conducted to evaluate the film morphologies. As exhibited in Fig. 3a (top right corner), the DPSO-modulated film exhibits a cracks/pinholes-free surface morphology compared to the pristine sample (top left corner). The surface morphologies of the achieved perovskite films in a wide field of vision are also shown in Fig. S4. Besides, the DPSO-modulated perovskite film demonstrates a tight and compact morphology across the crystals, as concluded from the cross-sectional SEM images (Fig. 3a, down row), which is beneficial for the carrier transport and the device performance. These phenomena can be ascribed to the regulated crystallization process. Then, time-of-flight secondary-ion mass spectrometry (ToF-SIMS) was performed to reveal the distribution of DPSO molecules across the perovskite film. As depicted in Fig. 3b, characteristic elements for the vertical functional layers underlying the perovskite film were recorded as a function of the etching time. It can be found that the O^−^ and S^−^ are distributed to the superficial zone near the surface of the perovskite film, which is proposed to be beneficial for manipulating the surface trap states in Sn–Pb perovskite film.

To detect the crystallinity of the as-prepared perovskite films, we performed X-ray diffraction (XRD) measurements. As exhibited in Fig. 3c, it is obvious that the XRD characteristic peak intensities corresponding to (100) and (200) planes are significantly enhanced for the DPSO-modulated perovskite film than the pristine sample, indicating an improvement of the film crystallinity. The improved film crystallinity is ascribed to the regulated crystallization process. Besides, it is widely acknowledged that the contact between the perovskite light harvester and the adjacent ETL has a critical impact on carrier transport. To observe the surface roughness of the perovskite films, atomic force microscopy (AFM) measurements were employed. The DPSO-modulated perovskite film exhibits a relatively flat and smooth surface morphology (Fig. 3d) with a lower root-mean-square (RMS) of 32.1 nm than the pristine film (41.6 nm). In addition, UV–Vis–NIR absorption spectra of the perovskite films were conducted to characterize their light harvest and optical bandgap. As illustrated in Fig. 3e, the optical absorption of DPSO-modulated perovskite film is improved in a broad spectrum range from 600 to 1000 nm compared to that of the pristine sample, which is attributed to the enhanced film crystallinity. Moreover, the optical bandgap of as-fabricated perovskites can be extracted from the Tauc plots, as shown in Fig. S5. It can be found that the bandgaps of both samples are ~1.26 eV, which is beneficial for the NIR responses of the photodetectors. Additionally, the steady-state photoluminescence (PL) spectra were collected to check the optical quality of the as-fabricated films. As demonstrated in Fig. 3f, the PL intensity of the DPSO-modulated film is higher than the counterpart of the pristine sample. In addition, a notable blueshift has been detected for the target film compared to the pristine sample, indicating a suppressed defect-mediated carrier nonradiative recombination. Furthermore, the quality uniformity of the prepared films in a relatively large range (50 μm × 50 μm) was further confirmed by PL mapping. As depicted in Fig. 3g, the DPSO-modulated perovskite film is more homogeneous than the pristine sample, which can be attributed to the regulated crystallization process.

Beyond that, the optical properties of the prepared Sn-Pb perovskite films were evaluated by time-resolved photoluminescence (TRPL) spectra. As exhibited in Fig. 3h, the target sample exhibits a longer PL decay lifetime (477.84 ns, Table S1) than the pristine perovskite film (111.67 ns). The prolonged average carrier lifetime confirms the suppressed charge nonradiative recombination. Besides, the TRPL spectra for perovskite films with different carrier quenching layers (poly[bis(4-phenyl) (2,4,6-trimethylphenyl) amine] (PTAA) for the hole and fullerene (C_60_) for the electron quenching, respectively) were also performed (Fig. S6 and Table S2) to evaluate the carrier diffusion length. As exhibited in Fig. 3i, the DPSO-modulated perovskite film exhibits longer electron (1993 nm) and hole diffusion lengths (1559 nm) than the pristine film (886 nm for electron and 586 nm for hole diffusion length, respectively), which is consistent with the longer carrier lifetime analyzed above. It also suggests a reduced trap state density in the DPSO-modulated perovskite film and the associated heterojunctions.

To quantify the impact of DPSO molecule incorporation on the trap state density (*N*_trap_) of the prepared Sn–Pb perovskite films, we further carried out the space charge limited current (SCLC) plots based on the hole-only and electron-only devices. The hole-only device with an architecture of indium-doped tin oxide (ITO)/nickel oxide (NiO_*x*_)/Sn–Pb perovskite/PTAA/silver (Ag) is illustrated in Fig. S7a. The *N*_trap_ of DPSO-modulated film was calculated to be 1.0 × 10^16^ cm^−3^ (Table S3), which was lower than the pristine sample (1.29 × 10^16^ cm^−3^). The reduced hole trap density is ascribed to the suppressed oxidation of Sn^2+^ and the reduced self-*p*-doping. The electron-only device with a representative structure of ITO/tin oxide (SnO_2_)/Sn–Pb perovskite/[6,6]-phenyl-C61-butyric acid methyl ester (PCBM)/Ag is diagrammed in Fig. S7b. The *N*_trap_ of DPSO-modulated perovskite film was calculated to be 8.05 × 10^15^ cm^−3^, which was lower than the pristine sample (1.35 × 10^16^ cm^−3^). The reduced electron trap density is attributed to the suppression of the undercoordinated Pb^2+^ and Sn^2+^ defects.

In addition, the femtosecond transient absorption spectroscopy (TAS) and transient photovoltage (TPV) technologies were comparatively adopted to investigate the charge carrier transport dynamics within perovskite films. The TAS of perovskite films was conducted with a pump excitation wavelength of 515 nm and probed in a range from 750 to 1000 nm. As shown in Fig. 3j, a stronger ground-state bleaching (GSB) signal has been detected at a probe wavelength around 940 nm in the DPSO-modulated Sn–Pb perovskite film than that of the pristine sample. The generation of the GSB signal has been acknowledged to the electrons in the excited state and holes in the ground state for the reduction of absorbing the probe photons during the corresponding transitions^34^. From the TAS at various time delays produced after the pump excitation for the pristine and DPSO-modulated perovskite films (Fig. 3k), it can be found that the latter exhibited a slowed decay profile, which implies a longer carrier lifetime and a suppressed carrier recombination^9^. Besides, they can also be confirmed by the TPV results. As shown in Fig. S8, the charge carrier recombination lifetime (1.5 μs) of the DPSO-modulated device is longer than that of the pristine device (910 ns), indicating a reduced carrier recombination after introducing DPSO.

### Performance of Sn–Pb perovskite NIR photodetectors

**Fig. 4:**
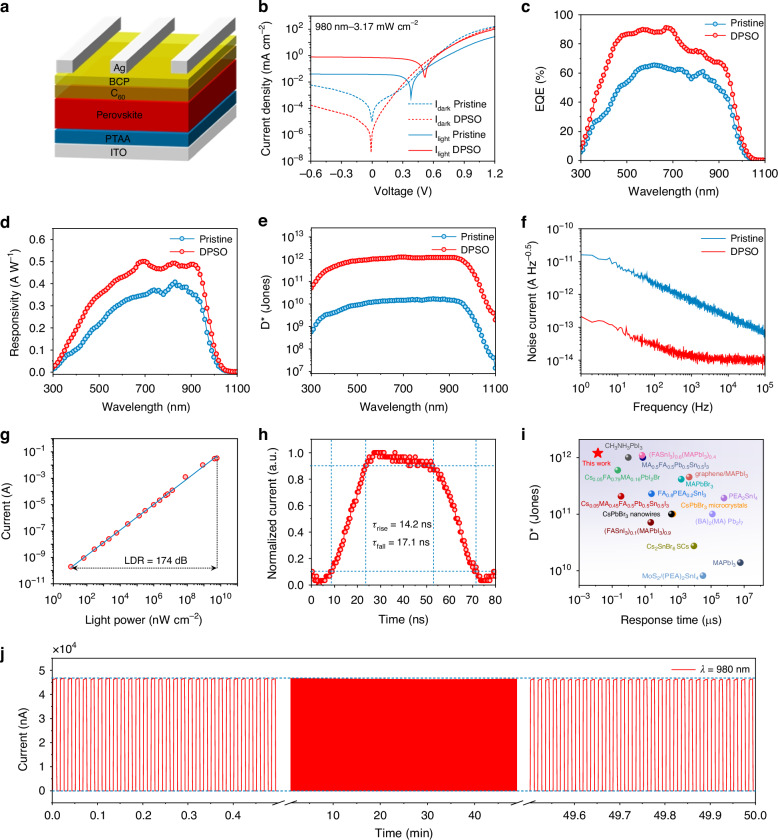
Architecture and performance of the single-pixel Sn–Pb perovskite photodetector. **a** Structure diagram of the photodetector. **b** Current density–voltage characteristics of the fabricated photodetector under dark and 980 nm illumination. **c** EQE, **d** responsivity, **e** Specific detectivity of the photodetectors operated at zero bias. **f** Noise spectral density of the photodetectors. **g** LDR, **h** Transient response of the optimal photodetector (DPSO-modulated). **i** Specific detectivity and response time statistics of the representatively reported perovskite photodetectors. **j** Continuous on/off response current tracking of the optimal photodetector at the wavelength of 980 nm under zero bias for 50 min

Inspired by the remarkable properties of the as-prepared Sn–Pb perovskite films, the self-powered NIR photodetectors with a vertical stack structure of ITO/PTAA/Sn–Pb perovskite/C_60_/bathocuproine (BCP)/Ag were manufactured (Fig. 4a). The cross-sectional SEM image of the DPSO-modulated device is shown in Fig. S9. Note that the fabricated device demonstrates a clear-cut and close-touch interface. Subsequently, their representative metrics were evaluated. As shown in Fig. 4b, the dark current density (*J*_d_) of the DPSO-modulated device is 4.7 × 10^−8^ mA cm^−2^ at zero bias, while the pristine device exhibited a *J*_d_ of 9.6 × 10^−6^ mA cm^−2^. The significantly suppressed dark current density is ascribed to the decreased leak current owing to the pinhole-free perovskite films and the suppressed trap-assisted carrier recombination. Moreover, the DPSO-modulated device exhibits a significantly higher photocurrent irradiated at a representative wavelength of 808, 980 and 1064 nm (Fig. S10) than the pristine one, indicating an efficient carrier transport. It can be ascribed to the improved energy band alignment of perovskite films with the adjacent charge transport layers (Fig. S11). External quantum efficiency (EQE) spectra of the fabricated devices are exhibited in Fig. 4c. Notably, the DPSO-modulated device exhibits an impressive EQE response at the wavelength of 300–1000 nm under zero bias, which is significantly higher than the counterpart of the pristine device. This result can be ascribed to the efficient carrier transport and photocurrent extraction, and suppressed carrier recombination induced by the homogeneous and smooth surface morphology, improved crystallinity, and reduced trap density of the DPSO-modulated perovskite film.

To figure out the charge carrier transport dynamics in the photodetectors, the built-in potential of the devices was comparatively analyzed by the Mott-Schottky plots. As depicted in Fig. S12, the DPSO-modulated device demonstrates a higher built-in potential than the pristine sample, which is responsible for the efficient charge carrier separation and transport, as well as the photocurrent response. In addition, it can be found that the DPSO-modulated device demonstrates a peak responsivity of 0.49 A W^−1^ in the NIR region (780–1100 nm) at zero bias (Fig. 4d), suggesting an excellent NIR response. As exhibited in Fig. 4e, the DPSO-modulated device demonstrated a higher peak specific detectivity of 1.20 × 10^12^ Jones than the pristine device (1.71 × 10^10^ Jones) at the wavelength of 830 nm. Besides, the noise current spectra of the fabricated devices are exhibited in Fig. 4f. It can be found that the total noise of the DPSO-modulated device is apparently lower than that of the pristine device, which is beneficial for the specific detectivity and LDR. As shown in Fig. 4g, the LDR of the manufactured target photodetectors is 174 dB, which is dramatically higher than the reported results of silicon photodiodes^35^. The large LDR implies that our fabricated photodetector has the potential to detect weak light signals.

The response speed is considered one of the most important parameters for photodetectors, and it is generally evaluated by the response time, including the rise and fall time^36^. As shown in Fig. 4h, the optimal self-powered Sn–Pb perovskite photodetector achieved a rise time (*τ*_rise_) of 14.2 ns and a fall time (*τ*_fall_) of 17.1 ns, indicating a fast response to the modulated optical signal. The multicycle signal outputs, including the modulated optical signal and device response, are demonstrated in Fig. S13. The performance of our photodetector is comparable with the commercial photodiodes in terms of the response speed and the specific detectivity (Table S4). Note that our device demonstrates an ultrafast response speed along with a top-level specific detectivity among the representative reported perovskite photodetectors. The representative results are compared in Fig. 4i and Table S5. Besides, the frequency response (Fig. S14) indicates that the prepared photodetector demonstrates an impressive −3 dB bandwidth of 11.4 MHz.

To evaluate the operational stability of the photodetector, the on/off current output of the DPSO-modulated devices without any encapsulation in ambient air under the NIR irradiations was recorded for 50 min. As shown in Fig. 4j, it can be found that the photocurrent (*λ* = 980 nm, *P*_in_ ≈ 2.89 mW cm^−2^) and dark current of the photodetector demonstrate negligible attenuation during the operational process with a relatively large on/off ratio over 10^4^. In addition, it also exhibited a stable output under the irradiation of 808 nm (*P*_in_ ≈ 1.20 mW cm^−2^) and 1064 nm (*P*_in_ ≈ 8.33 mW cm^−2^), as shown in Fig. S15. These results suggest that our prepared NIR photodetectors can be stably operated in ambient air, which has great potential in the application of some intelligent consumer electronics.

To estimate the imaging ability of the manufactured photodetectors, we fabricated 5 × 5 photodetector arrays with a single-pixel size of 3 mm × 3 mm. The schematic illustration and photograph of the designed arrays, as well as the imaging measurement system, are shown in Figs. S16 and S17. The NIR radiations reach the arrays across a designed mask with letters of “S”, “X”, and “U”, and the output current was recorded in sequence. The imaging results are exhibited in Fig. S18. It can be concluded that the photodetector arrays demonstrate satisfactory imaging ability without external bias in the NIR region (*λ* = 808, 980, and 1064 nm).

### Monolithically integrated NIR imager

**Fig. 5:**
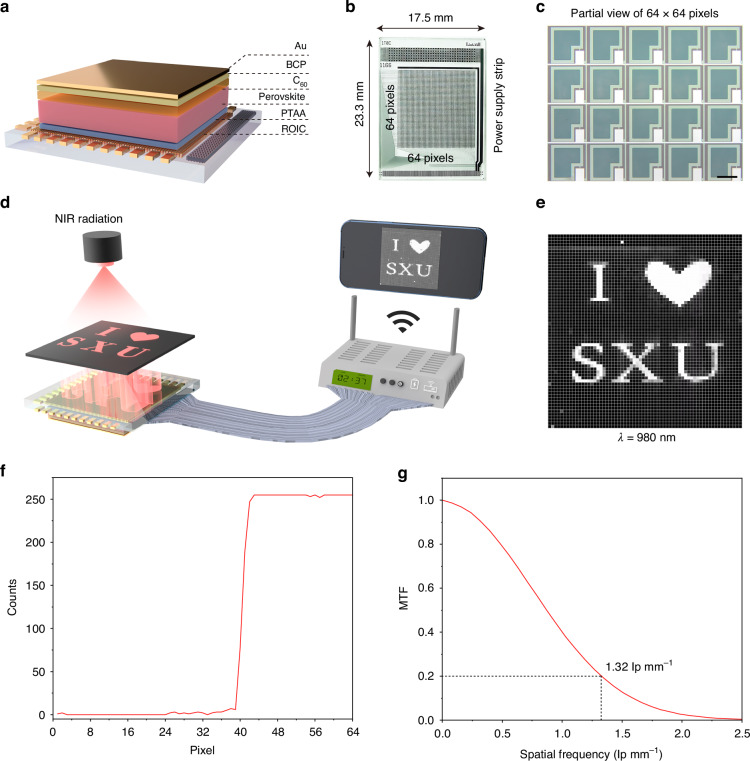
Monolithic integration and performance of the NIR imagers. **a** Schematic diagram of monolithically integrating the photodetector with the TFT readout circuit. **b** Photograph of the used TFT backplane along with its size and pixels. **c** Microphotograph of a partial view of the used TFT pixel arrays, the scalebar is 100 μm. **d** Schematic illustration of the NIR imaging system for the imagers. **e** Imaging of the imager for a designed pattern of “I ♥ SXU” operated at a wavelength of 980 nm (≈11.35 mW cm^−2^). **f** Image crosstalk as the function of a pixel row with a sharp metallic edge. **g** MTF of the NIR imager

With the seductive success of the fabricated photodetector for NIR radiation detection, it is highly desirable to be monolithically integrated with the well-established ROIC technologies for further application in imagers. Then, the optimal photodetector was monolithically integrated with the commercial a-Si TFT backplane in a vertical stack to construct the NIR imager, as shown in Fig. 5a. To achieve better electrical contacts, gold (Au) electrodes were used for the manufactured imagers. Notably, all the process procedures were sequentially operated at low temperatures (<150 °C), providing a great potential to fabricate flexible NIR imagers. The photograph of the commercial TFT backplane with a typical size of 17.5 mm × 23.3 mm is depicted in Fig. 5b, and its active area is 12.8 mm × 12.8 mm (64 × 64 pixels). The top-view optical microscopic image of partial TFT pixel arrays is exhibited in Fig. 5c, and the circuit diagram of a single pixel is demonstrated in Fig. S19. Particularly, ITO serves as an interconnection layer between the TFT backplane and the top-operated photodetector, and its cross-sectional schematic illustration is shown in Fig. S20.

To evaluate the imaging capability of the fabricated NIR imager, we first bonded it with the current readout system (Fig. S21). Then, a designed mask with the pattern of “I ♥ SXU” was covered on the top of the imager and illuminated by the 980 nm radiation. During the imaging process, the output current signals were read out line by line at a rate of 10.9 frames per second by the pulse row inside the matrix readout system. The output signals can be transmitted to a mobile phone and recorded by a wireless WIFI module, as illustrated in Fig. 5d. As exhibited in Fig. 5e and Supplementary video 4, a clear image with “I ♥ SXU” pattern was presented by the developed imager. The crosstalk between the pixels of the imager was measured by the slanted-edge method^37^. As shown in Fig. 5f, the signal output within one-line pixels was detected, and the maximum count was 255. Furthermore, the spatial resolution of our fabricated perovskite NIR imager was subsequently evaluated by the modulation transfer function (MTF) as line pairs per millimeter (lp mm^–1^). As depicted in Fig. 5g, the developed imager achieved 1.32 lp mm^–1^ at MTF = 0.2, while the pixel pitch was 200 μm. Besides, the fabricated imager can also be well operated under the illumination of 808 and 1064 nm (Fig. S22, Supplementary videos 5, 6). Based on the above analysis and demonstration, it can be concluded that our manufactured imager can be operated well in the NIR region.

## Discussion

In summary, we have developed a cost-effective and high-performance NIR imager by monolithically integrating the Sn–Pb perovskite photodetector with a commercial TFT readout circuit. The NIR photodetector has demonstrated a dark current density of 4.7 × 10^−8^ mA cm^−2^, specific detectivity of 1.20 × 10^12^ Jones, –3 dB bandwidth of 11.4 MHz, LDR of 174 dB, and rise/fall time of 14.2/17.1 ns. A 64 × 64 NIR imager with an impressive spatial resolution of 1.32 lp mm^−1^ at MTF of 20% has been achieved. The developed Sn–Pb perovskite NIR imager provides a promising avenue for integrating its application in smart consumer electronics.

## Materials and methods

### Materials

Formamidinium iodide (FAI, 99.99%), lead (II) iodide (PbI_2_, 99.99%), tin (II) iodide (SnI_2_, 99.999%), nickel oxide nanoparticles (NiO_*x*_, 10 nm), [6,6]-phenyl-C61-butyric acid methyl ester (PCBM, 99.9%), and indium-doped tin oxide (ITO, 15 Ω sq^−1^) glasses were provided by Advanced Electronic Technology Co., Ltd., China. Tin (IV) oxide solution (SnO_2_, 15% in H_2_O colloidal dispersion) was purchased from Alfa Aesar. Tin (II) fluoride (SnF_2_, 99%) was purchased from Aladdin, China. N,N-dimethylformamide (DMF, 99.8%), dimethyl sulfoxide (DMSO, 99.7%), chlorobenzene (CB, 99.8%), and isopropanol (IPA, 99.5%) were purchased from J&K, China. Tin powder (≥99.5%) was purchased from Mackli, China. Methylammonium iodide (MAI, 99.5%), C_60_ (99.0%), and Poly[bis(4-phenyl) (2,4,6-trimethylphenyl) amine] (PTAA, 6000–15,000) were obtained from Xi’an Yuri Solar Co., Ltd, China. Toluene (≥99.5%) was purchased from Sinopharm Chemical Reagent Co., Ltd., China. Diphenyl sulfoxide (DPSO, >99.0%) and bathocuproine (BCP, >99.0%) were purchased from Tokyo Chemical Industry Co., Japan. High-purity silver and gold particles were purchased from commercial sources. All chemicals were directly used as received without any further purification unless otherwise specified. Thin-film transistor (TFT) backplanes were provided by LinkZill Technology Co., Ltd., China.

### DFT calculation

The density-functional theory (DFT) calculations were performed using the Vienna Ab initio Simulation Package (VASP). The projected augmented wave (PAW) method and the Perdew–Burke–Ernzerhof (PBE) exchange-correlation functional within the generalized gradient approximation (GGA) were used to obtain the lattice constants. An energy cutoff of 500 eV was set for the plane-wave function’s expansion. The van der Waals (vdW) dispersion correction was found necessary to yield more accurate lattice constants, which were described by the DFT-D3 correction. The primitive unit cell structures of cubic-phase FASnI_3_ were obtained by applying Monkhorst–Pack sampling with a Γ-centered 6 × 6 × 6 *k*-point grid. A supercell slab of 3 × 3 × 1 FASnI_3_ [001] surface was used in the simulation. The adsorption of O_2_ and O_2_ + DSPO was performed on the FASnI_3_ [001] surface, respectively. The slabs with different surfaces were separated by a vacuum spacing of >15 Å to avoid the interaction between period slabs. In the surface optimization, the top three atom layers were free to relax, while the rest atoms were fixed during the calculation. Then, the Brillouin zone of FASnI_3_ [001] slabs was sampled by a 1 × 1 × 1 *k*-mesh. In all geometric optimization, the residual force was converged until the total energy changes were <1.0 × 10^−5^ eV and the maximum force component acting on each atom was <0.01 eV Å^−1^.

### Sn–Pb perovskite precursor ink preparation

1.1 M Sn–Pb perovskite precursor solution with a representative composition of FA_0.7_MA_0.3_Sn_0.5_Pb_0.5_I_3_ was prepared for the construction of photodetectors and imagers. Typically, the precursors of FAI (0.1324 g), MAI (0.0524 g), SnI_2_ (0.2049 g), PbI_2_ (0.2536 g), DPSO (0.0044 g), and SnF_2_ (0.0086 g) were dissolved into the mixed solvent of DMSO and DMF (volume ratio of 1:4). The precursor solutions were stirred for 2 h at room temperature. Tin (Sn) powder was also added to the precursor solutions to further restrain the Sn^2+^ oxidation. All solutions were filtered by a 0.22 μm membrane before use.

### Device fabrication

ITO glass substrates were firstly scribed by a nanosecond pulsed laser (355 nm, 3 W). The etched ITO glasses were then ultrasonically cleaned with detergent, deionized water, ethanol, acetone, and isopropanol for 20 min, respectively. The washed ITO substrates were subsequently dried at 100 °C in an oven overnight. After that, they were further cleaned with the UV-ozone (UVO) for 20 min and transferred into a N_2_-filled glovebox before use. PTAA dissolved in toluene at a concentration of 2 mg mL^−1^ was spin-coated on the cleaned ITO substrates at 5000 r.p.m. for 30 s and annealed at 100 °C for 10 min. Then, the prepared perovskite precursor ink was dropped on the top of PTAA with a typical two-step spin-coating procedure at 1000 r.p.m. for 10 s and 5000 r.p.m. for 30 s, and then annealed at 100 °C for 10 min. Finally, C_60_, BCP, and Ag (Au) electrodes were consecutively deposited by thermal evaporation at a vacuum of <5.0 × 10^−4^ Pa, and the evaporation rate was controlled at 0.1 Å s^−1^ for the thickness of 28, 6, and 100 nm, respectively.

### Fabrication of samples for carrier diffusion and SCLC characterizations

#### Samples for carrier diffusion length characterizations

The quartz glass substrates were cleaned, dried, and UVO treated by the same procedures for washing ITO glasses. Subsequently, the PTAA hole quenching layer, Sn–Pb perovskite films, and C_60_ electron quenching layer were prepared with the same method and procedures of fabricating the corresponding layers for devices. Then, the achieved samples of glass/PTAA/perovskite, glass/perovskite, and glass/perovskite/C_60_ were, respectively, used to carry out time-resolved photoluminescence (TRPL) measurements for the assessment of ${\tau }_{{\rm {Q}}}^{{\rm {h}}}$, ${\tau }_{0}$ and ${\tau }_{{\rm {Q}}}^{{\rm {e}}}$ and calculating carrier diffusion length by the method analyzed in the following.

#### Samples for SCLC characterizations

NiO_*x*_ nanoparticles dispersed in deionized water at a concentration of 10 mg mL^−1^ were spin-coated onto the cleaned ITO substrate at 4000 r.p.m. for 30 s, and subsequently annealed at 150 °C for 30 min to construct a hole-only device. SnO_2_ solution diluted by deionized water (volume ratio was 1:8) was spin-coated onto the prepared ITO substrate at 4000 r.p.m. for 30 s, and subsequently annealed at 150 °C for 30 min. PCBM solution (20 mg mL^−1^ in chlorobenzene) was spin-coated on perovskite film at 3000 r.p.m. for 10 s, and subsequently annealed at 70 °C for 10 min. SnO_2_ and PCBM films were used to fabricate the electron-only device. Unless otherwise specified, other materials, including ITO substrate, PTAA, perovskite film and the silver electrode, were used with the same method and procedures for the fabrication of photodetectors described above.

### Characterizations

In-situ ultraviolet–visible (UV–vis) absorption spectra of perovskite precursor inks and UV–vis–NIR spectra of perovskite films were obtained by using a spectrophotometer (UNIC 3802, China). X-ray photoelectron spectroscopy (XPS) and ultraviolet photoelectron spectroscopy (UPS) were performed by a K-Alpha + spectrometer (K-Alpha, Thermo Scientific). Dynamic light scattering (DLS) of the perovskite precursor solutions was tested by a Zetasizer Nano ZS90 (Malvern). Top-view and cross-sectional morphologies of the achieved Sn–Pb perovskite films, and the cross-sectional morphologies for the manufactured photodetectors were observed by field-emission scanning electron microscopy (FE-SEM, Hitachi SU 8010, Japan). Time-of-flight secondary ion mass spectrometry (ToF-SIMS) measurements were carried out by a double-beam focused ion microscope (TESCAN-S8000). The crystal structures of the prepared Sn–Pb perovskite films were detected by X-ray diffraction (XRD) equipped with a diffractometer with Cu Kα radiation (D2 PHASER, Bruker). The roughness and surface morphologies of the prepared Sn–Pb perovskite films were characterized by Bruker Dimension Icon atomic force microscopy (AFM). Fourier transform infrared (FTIR) spectra were recorded by a Vertex 80v spectrometer (Bruker, Germany). Steady-state photoluminescence (PL) and the mapping images were collected by an MS starter 100 microscopic spectroscopy system equipped with a 532 nm laser for excitation (METATEST Corporation, China). TRPL spectra were recorded by a single-photon detector and analyzed by a single-photon counting module (TCSPC, HydraHarp 400, PicoQuant). The Mott–Schottky characterization was performed on an electrochemical workstation (CHI 760E, China). Transient absorption spectra (TAS) of the fabricated Sn–Pb perovskite films were characterized by the ultrafast transient absorption spectrometer (TIME-TECH SPECTRA). Transient photovoltage (TPV) decay was carried out by a homemade transient photoelectric test system coupled with a function waveform generator (SDG 1022X) and MSO24 mixed signal oscilloscope (Tektronix). The dark current density and space charge limited current (SCLC) results were collected by a highly accurate source meter (2602B, Keithley) in dark conditions. The noise current spectra were performed at zero bias by a semiconductor parameter analyzer (FS-Pro, Primarius) equipped with a noise analysis module (HNZ-A). External quantum efficiency (EQE) was tested by a photodetector measurement system (FineDet 900, China) equipped with a xenon lamp, a monochromator, and a standard silicon detector for calibration. The performance of the fabricated perovskite NIR imagers was carried out by the readout system (LinkZill, China).

The carrier diffusion length has been calculated by the following equation:

1 $$\frac{{{L}}_{{\rm{D}}}}{{L}}=\frac{2}{{\uppi }}\sqrt{\frac{{{\tau }}_{0}}{{{\tau }}_{{\rm{Q}}}}-1}$$

where *L*_D_ is the carrier diffusion length, *L* is the thickness of perovskite film, *τ*_Q_ and *τ*_0_ are the lifetimes of perovskite films with and without the carrier quenching layers, respectively. In the context, ${\tau }_{{\rm {Q}}}^{{\rm {h}}}$ and ${\tau }_{{\rm {Q}}}^{{\rm {e}}}$ represents the lifetime of perovskite film with hole and electron quenching layer, respectively. ${L}_{{\rm {D}}}^{{\rm {h}}}$ and ${L}_{{\rm {D}}}^{{\rm {e}}}$ are the calculated hole and electron diffusion length, respectively.

The responsivity (*R*) has been calculated by Eq. (2):

2 $$R=\frac{{{{EQE}}}\times \lambda }{1240}$$

The specific detectivity (D*) of photodetectors has been calculated according to formula (3):

3 $${D}^{* }=\frac{R\sqrt{A\Delta f}}{{i}_{{\rm {{n}}}}}$$

where *R* represents the responsivity of the photodetector, *A* is the effective area of the device (0.09 cm^2^), Δ*f* is the electrical bandwidth (1 Hz), and *i*_n_ is the measured noise current.

LDR is commonly expressed with a logarithmic scale according to

4 $${\rm{LDR}}=201{\rm{g}}\frac{{I}_{\max }}{{I}_{\min }}$$

where *I*_max_ and *I*_min_ represent the maximum and minimum photogenerated current in the linear photoresponse range, respectively.

## Supplementary information

Supplementary Information for A monolithically integrated near-infrared imager with crystallization- and oxidation-modulated tin-lead perovskites

Natural crystallization process of the pristine Sn-Pb perovskite intermediate film

Natural crystallization process of the DPSO-modulated Sn-Pb perovskite intermediate film

Oxidation and aging process of SnI2 solution without and with DPSO

Imaging process of the fabricated perovskite NIR imgaer under 980 nm radiation

Imaging process of the fabricated perovskite NIR imgaer under 808 nm radiation

Imaging process of the fabricated perovskite NIR imgaer under 1064 nm radiation

## Data Availability

The data that support the findings of this study are available from the corresponding authors upon reasonable request.
