# Supplementary material for: A monolithically integrated near-infrared imager with crystallization- and oxidation-modulated tin-lead perovskites
Source: Light Sci Appl. 2025 Sep 4;14:304. doi: 10.1038/s41377-025-01987-8 (PMC12411627; doi:10.1038/s41377-025-01987-8)
Supplement: Supplementary file 1 — Supplementary Information for A monolithically integrated near-infrared imager with crystallization- and oxidation-modulated tin-lead perovskites [file 41377_2025_1987_MOESM1_ESM.docx]

**Supplementary Information for**

**A monolithically integrated near-infrared imager with crystallization- and oxidation-modulated tin-lead perovskites**

Zhichun Yang^1,2*^, Jingjing Liu^1,2^, Haotian Bao^1,2^, Zonghao Liu^3^, Zaiwei Wang^4^, Xiangdong Li^1,2^, Zhihao Chen^1,2^, Guofeng Zhang^1,2^, Ruiyun Chen^1,2^, Jianyong Hu^1,2^, Shuangping Han^5^, Wei Chen^3*^, Chengbing Qin^1,2*^, Liantuan Xiao^1,2,5*^, Suotang Jia^1,2^

^1^ State Key Laboratory of Quantum Optics Technologies and Devices, Institute of Laser Spectroscopy, Shanxi University, Taiyuan 030006, China.

^2^ Collaborative Innovation Center of Extreme Optics, Shanxi University, Taiyuan 030006, China.

^3^ Wuhan National Laboratory for Optoelectronics, Huazhong University of Science and Technology, Wuhan 430074, China.

^4^ Faculty of Materials Science and Energy Engineering, Shenzhen University of Advanced Technology, Shenzhen 518107, China.

^5^ College of Physics and Optoelectronic Engineering, Taiyuan University of Technology, Taiyuan 030600, China.

*Corresponding authors:

E-mail: [yangzhichun@sxu.edu.cn](mailto:yangzhichun@sxu.edu.cn); [wnlochenwei@hust.edu.cn](mailto:wnlochenwei@hust.edu.cn); [chbqin@sxu.edu.cn](mailto:chbqin@sxu.edu.cn); [xlt@sxu.edu.cn](mailto:xlt@sxu.edu.cn)

**Table of Contents**

Supplementary Note 1

Supplementary Note 2

Supplementary Figure 1-22

Supplementary Table 1-5

Supplementary Video 1-6

Supplementary References

**Supplementary Note 1. The average carrier lifetime of perovskite films without and with the charge** **quenching layers.**

The fitting of TRPL results was performed with the well-known bi-exponential function:

$Y=A_{1}\exp\left( \frac{-t}{\tau_{1}} \right)+A_{2}\left( \frac{-t}{\tau_{2}} \right)+y_{0}$ (1)

where *A*_1_ and *A*_2_ are the relative amplitudes, $\tau_{1}$ and $\tau_{2}$ are the lifetimes for the fast and slow recombination, respectively.

The average lifetime ($\tau_{ave}$) of Sn-Pb perovskite films without or with different carrier quenching layers (PTAA for the hole and C_60_ for the electron quenching) are calculated using the following equation:

$\tau_{ave}=\frac{A_{1}\tau_{1}^{2}+A_{2}\tau_{2}^{2}}{A_{1}\tau_{1}+A_{2}\tau_{2}}$ (2)

**Supplementary Note 2. Trap density of perovskite films.**

The trap densities of the prepared Sn-Pb films were characterized by the space charge-limited current (SCLC) technology. In this work, both the hole-only and electron-only devices were fabricated. Dark currents of the manufactured photodetectors were measured under a bias range of 0.01~1 V.

The trap densities were calculated by the equation 3:

$N_{t}=\frac{2\varepsilon_{0}\varepsilon V_{TFL}}{qL^{2}}$ (3)

where *ε*_0_ is the vacuum dielectric constant, *N*_t_ is the trap density in perovskite films, *ε* is the permittivity of perovskite film (54.87), *e* is the fundamental charge, and *L* is the thickness of the prepared perovskite films (416 nm for the pristine and 420 nm for the DPSO-modulated perovskite films extracted from the SEM results).


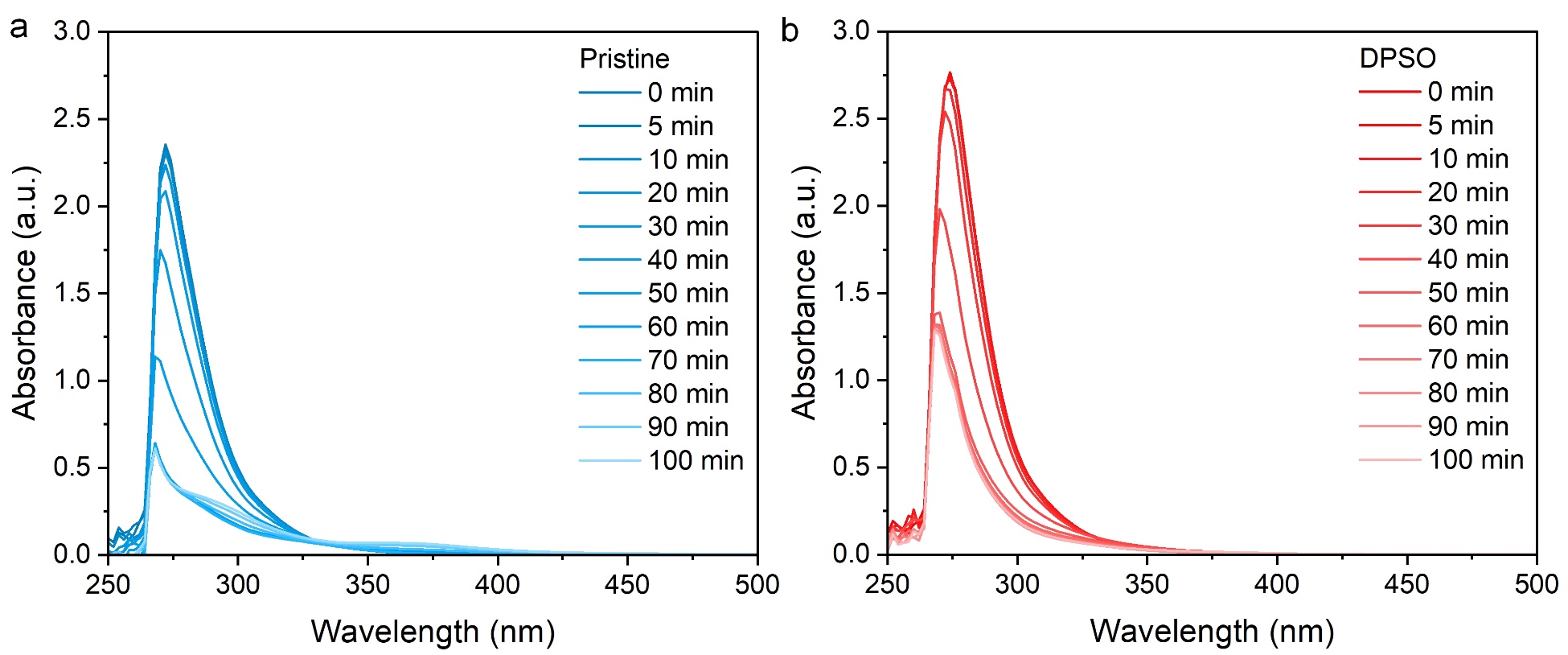


**Fig. S1 In-situ UV-vis absorption spectra of SnI_2_ solutions in ambient air. a,** SnI_2_ dissolved in DMF (Pristine). **b,** SnI_2_ dissolved in DMF**/**DPSO (named as DPSO).

**
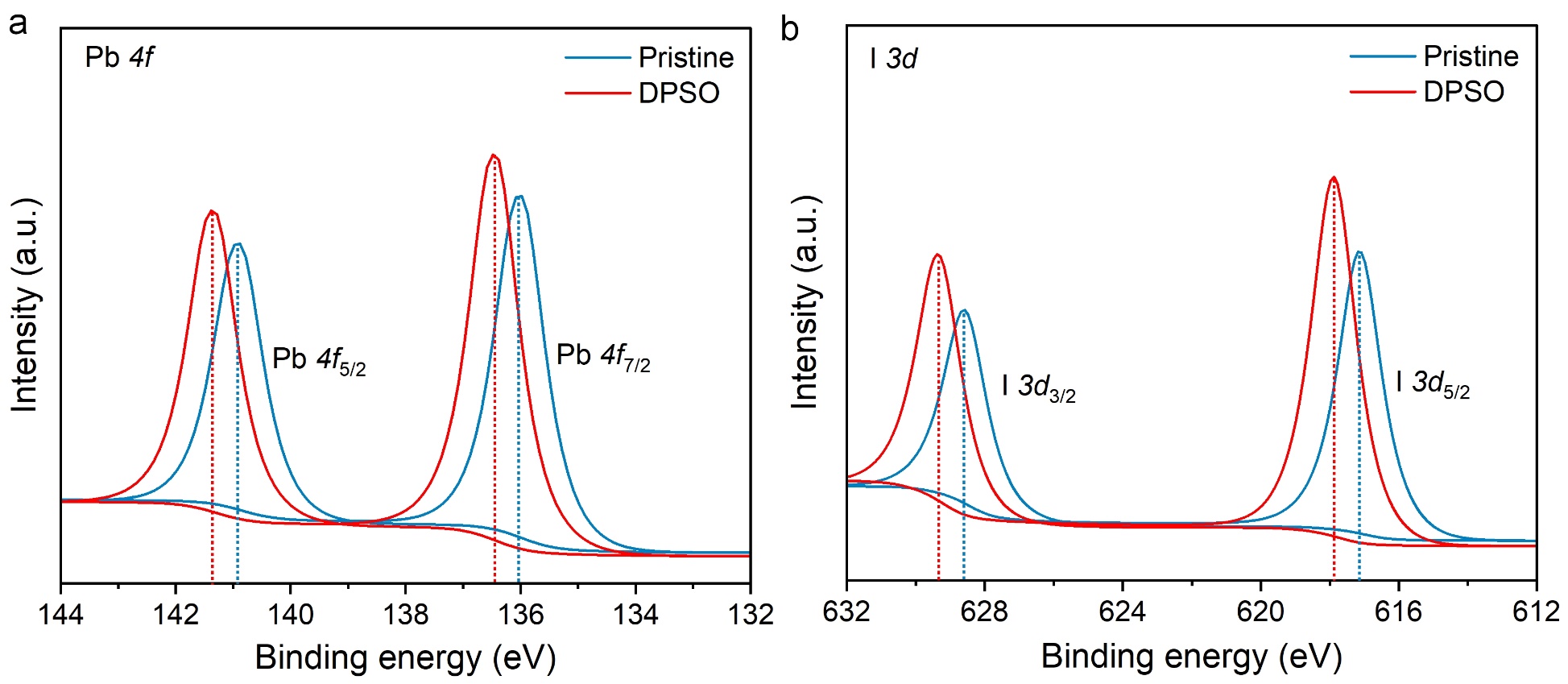
**

**Fig. S2 XPS of Pb 4*f* and I 3*d* for Sn-Pb perovskite films. a,** Pb 4*f*. **b,** I 3*d*.


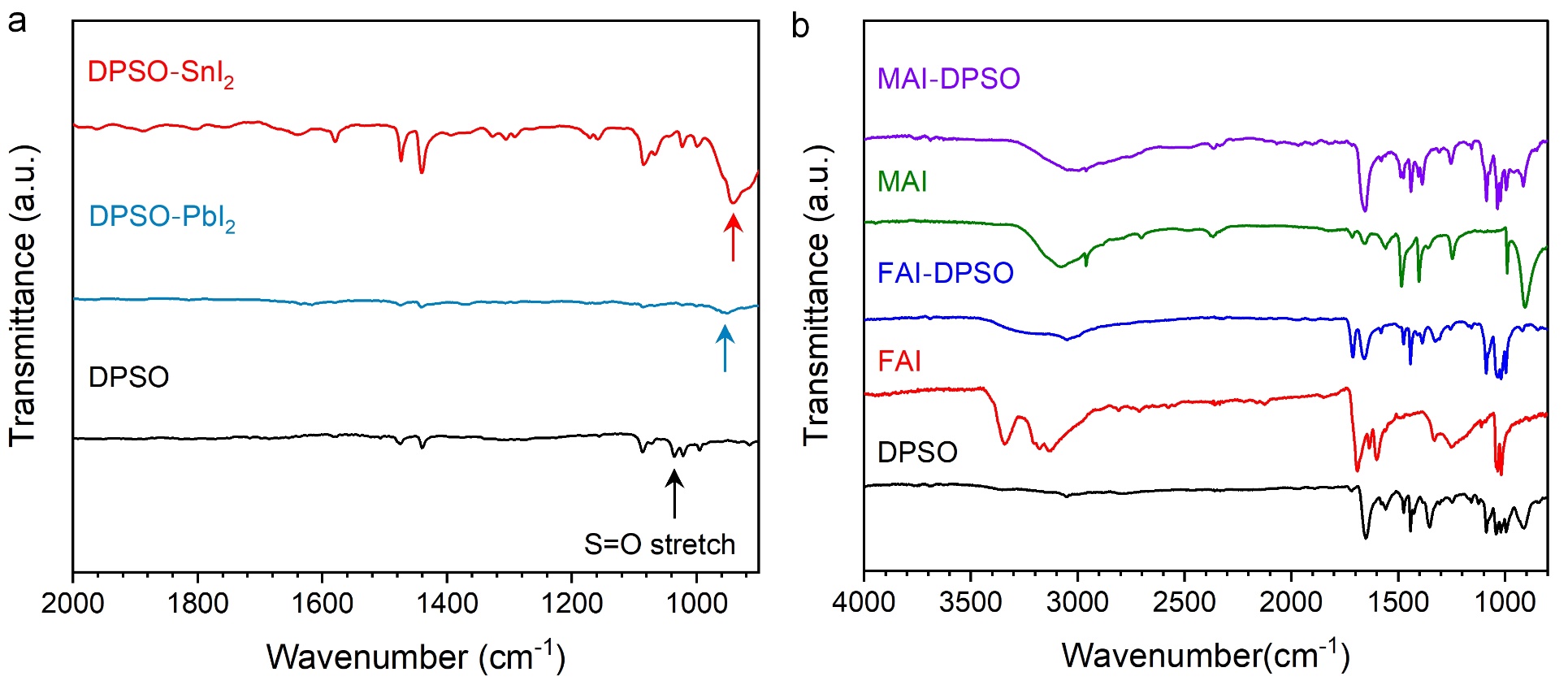


**Fig. S3 FTIR results of DPSO molecule and the related complexes. a,** FTIR results of bare DPSO, DPSO-PbI_2_ and DPSO-SnI_2_ powders. **b,** FTIR results of DPSO, FAI, DPSO-FAI, MAI, DPSO-MAI powders.


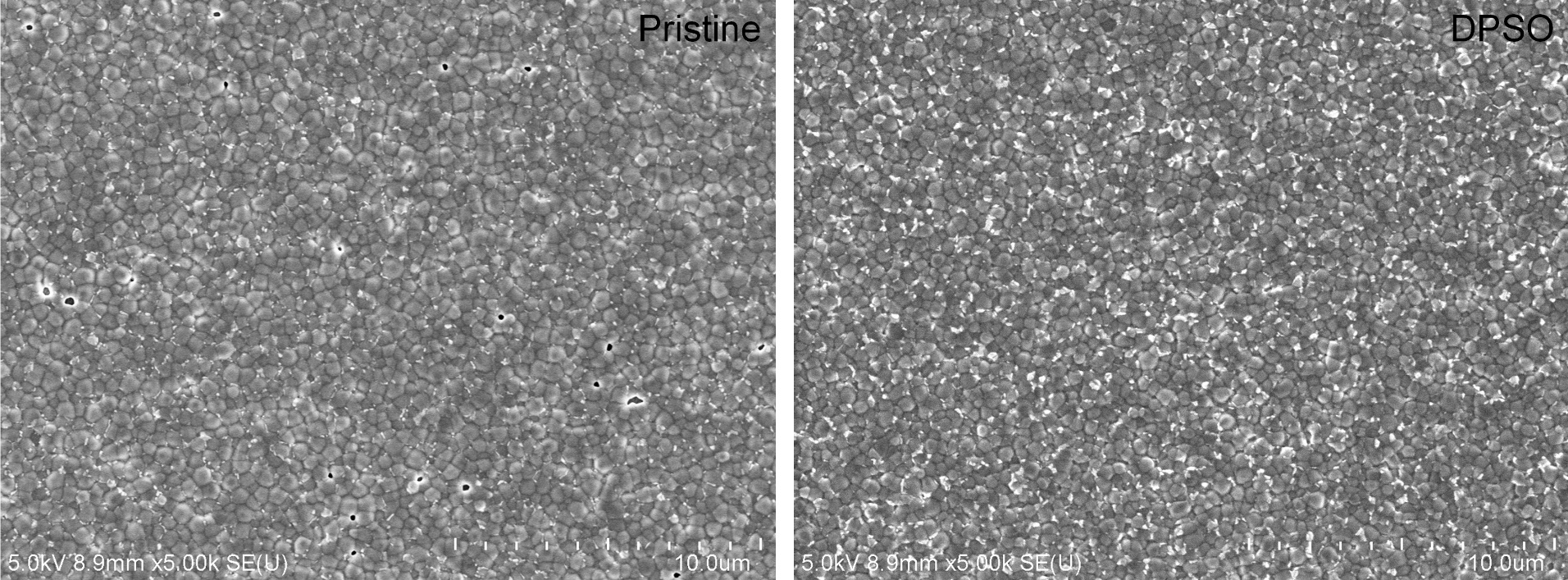


**Fig. S4** SEM images of Sn-Pb perovskite films.


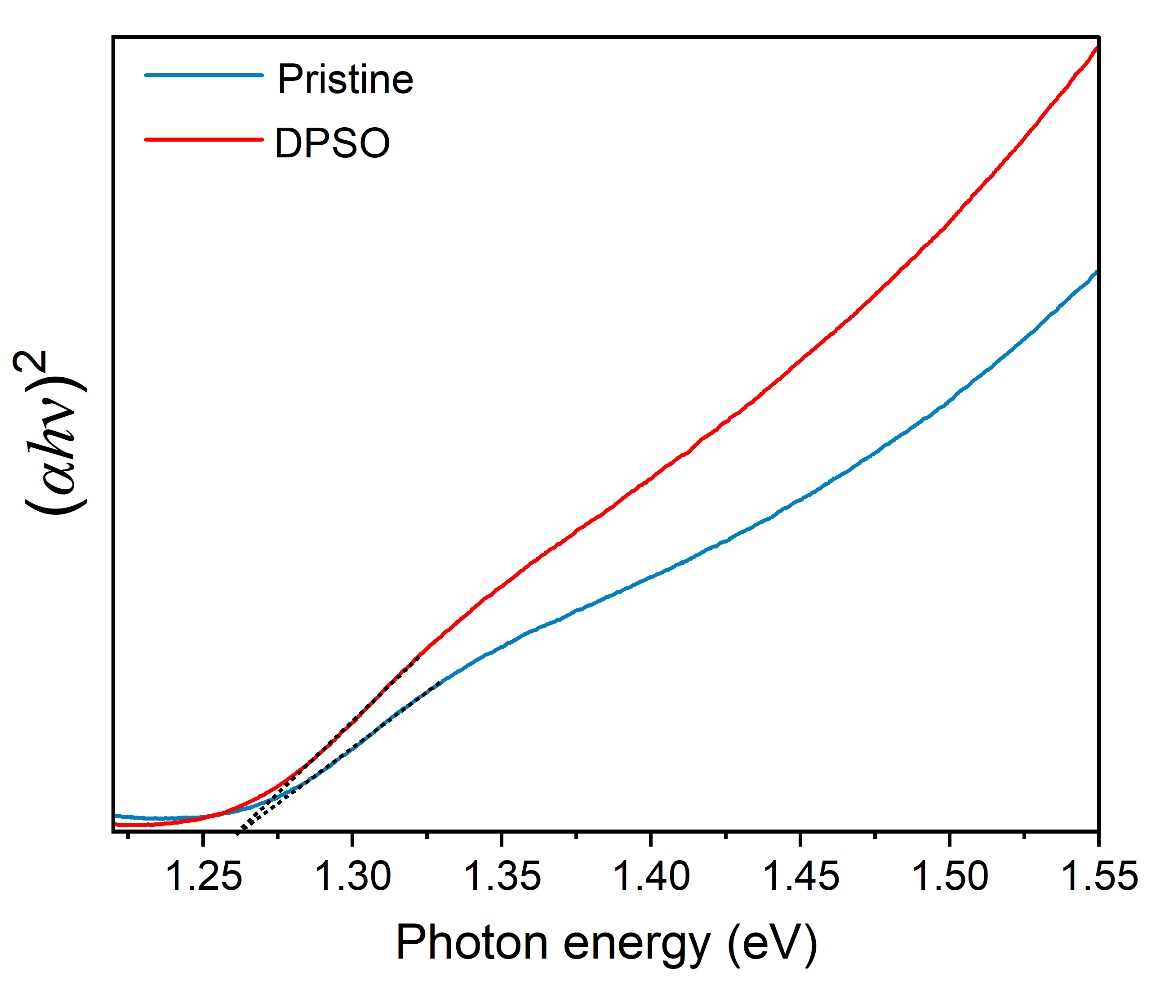


**Fig. S5** Tauc-plots of the prepared Sn-Pb perovskite films.

**
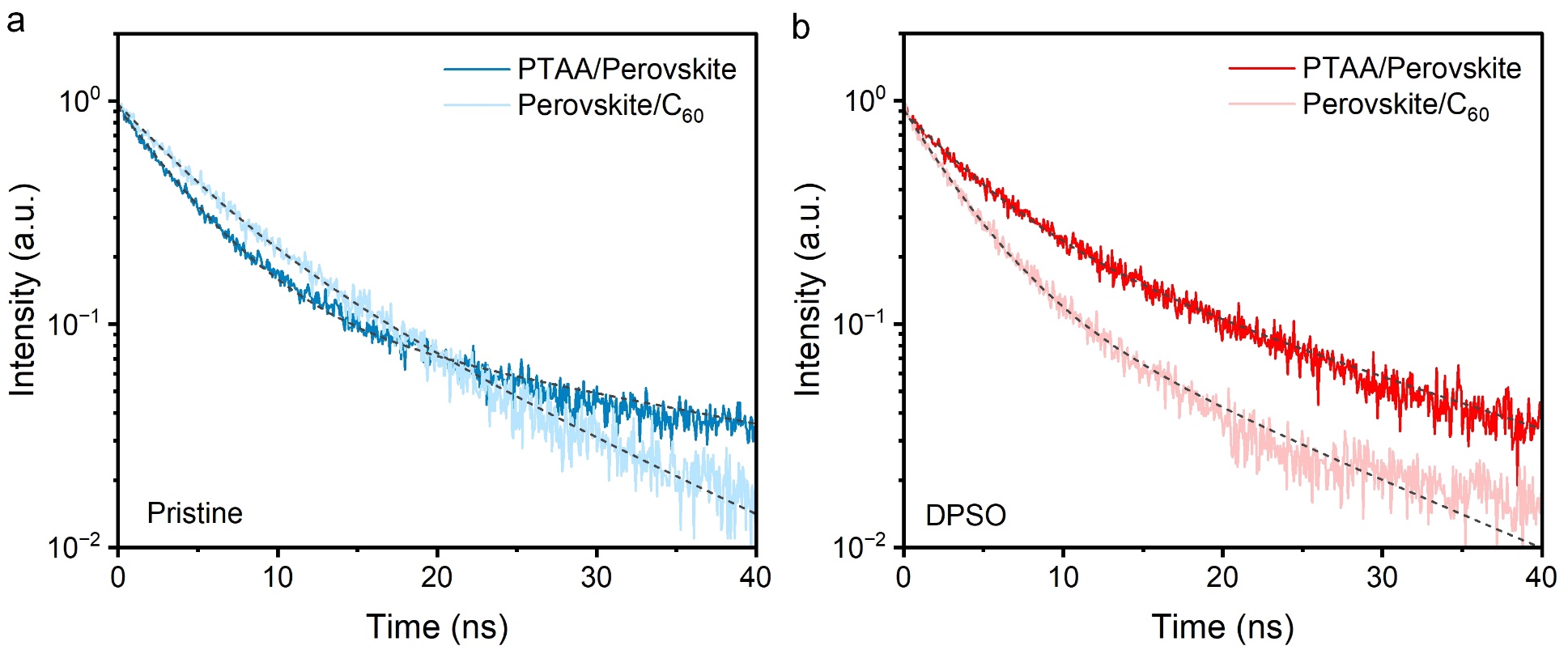
**

**Fig. S6 TRPL spectra of the prepared Sn-Pb perovskite films with different carrier quenching layers. a,** Pristine samples. **b,** DPSO-modulated samples.


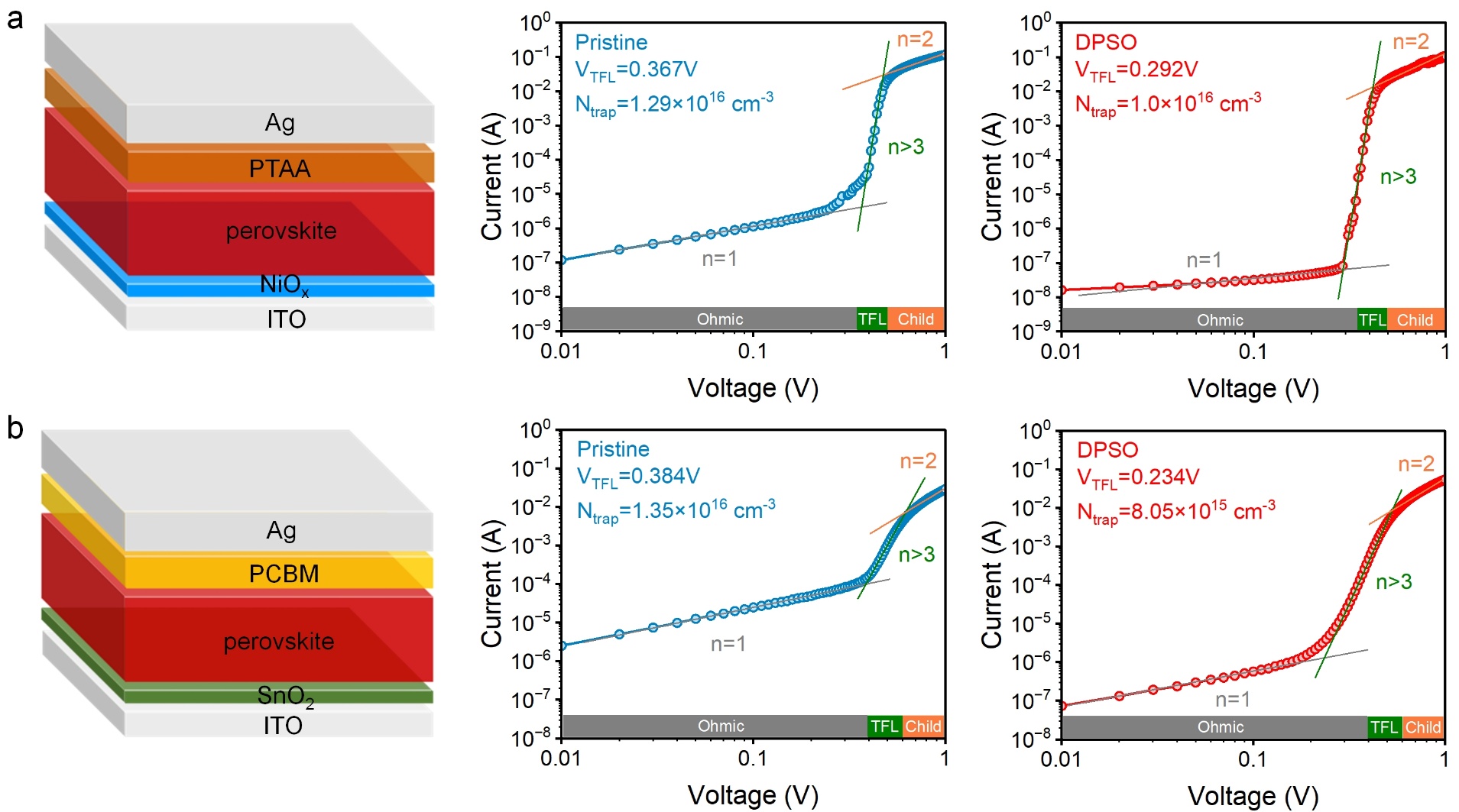


**Fig. S7 SCLC of the hole-only and electron-only devices fabricated with the pristine and DPSO-modulated perovskite films. a,** Hole-only devices. **b,** Electron-only devices.


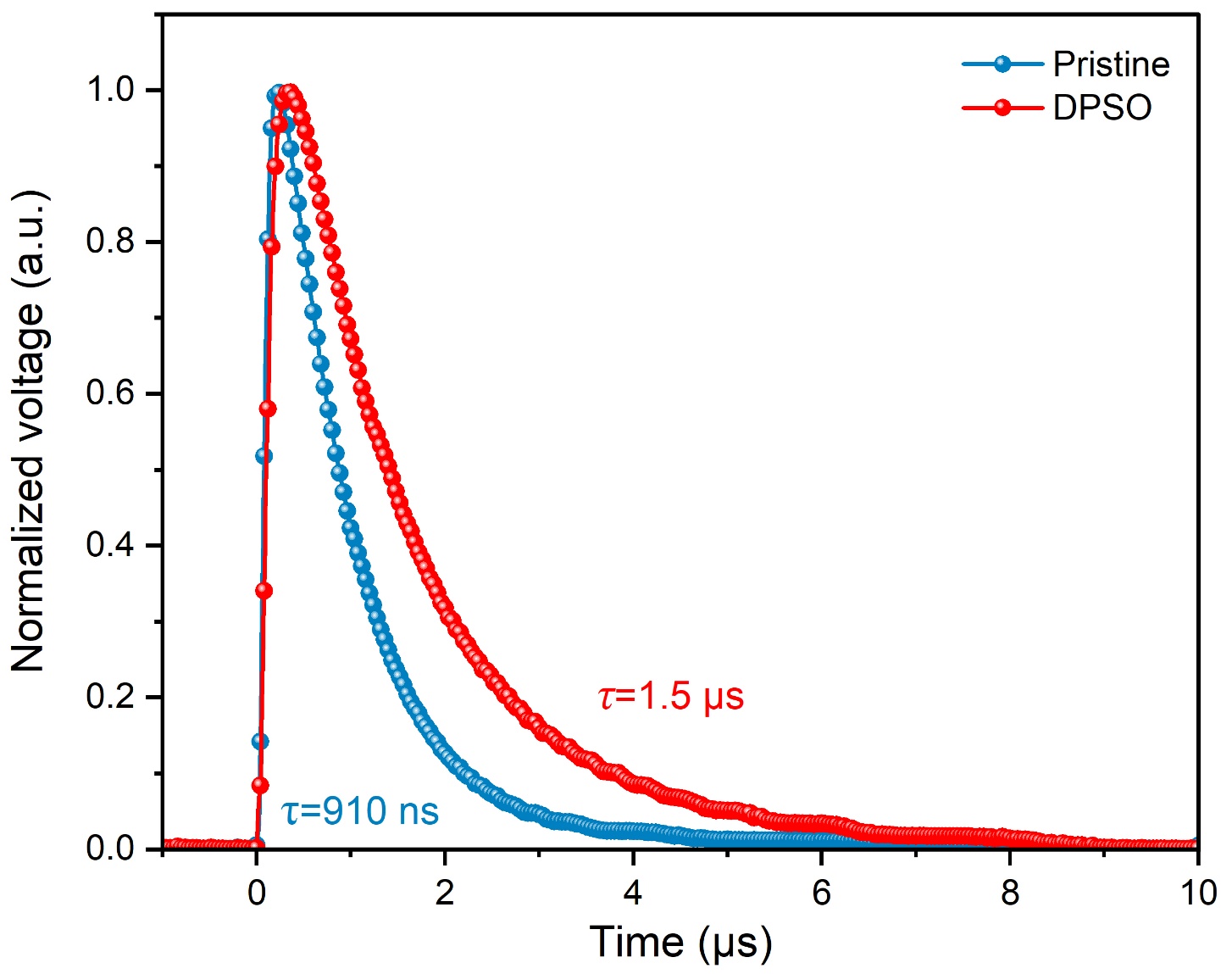


**Fig. S8** TPV curves of the devices with different perovskite films.


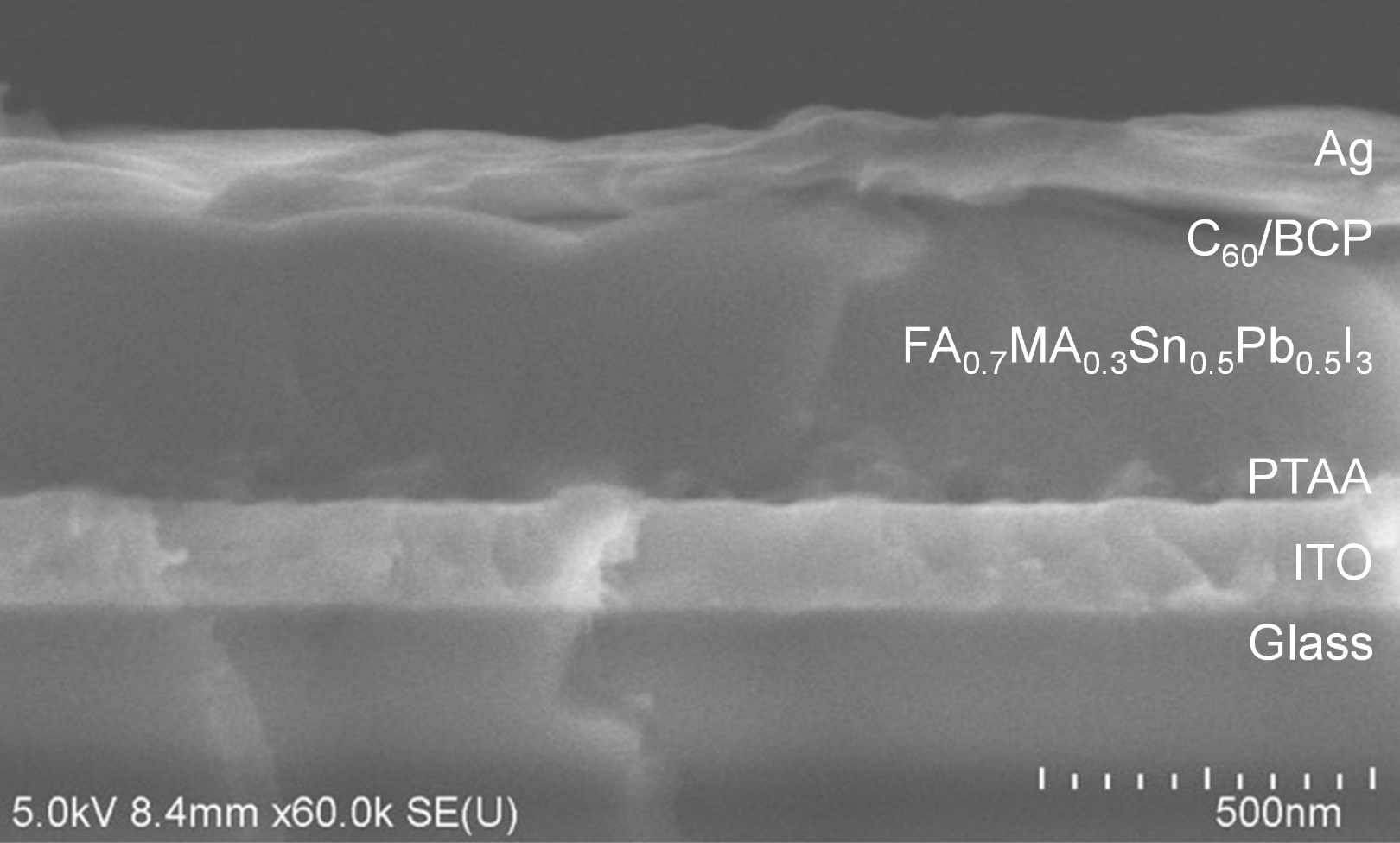


**Fig. S9** Cross-sectional SEM image of the DPSO-modulated photodetector.


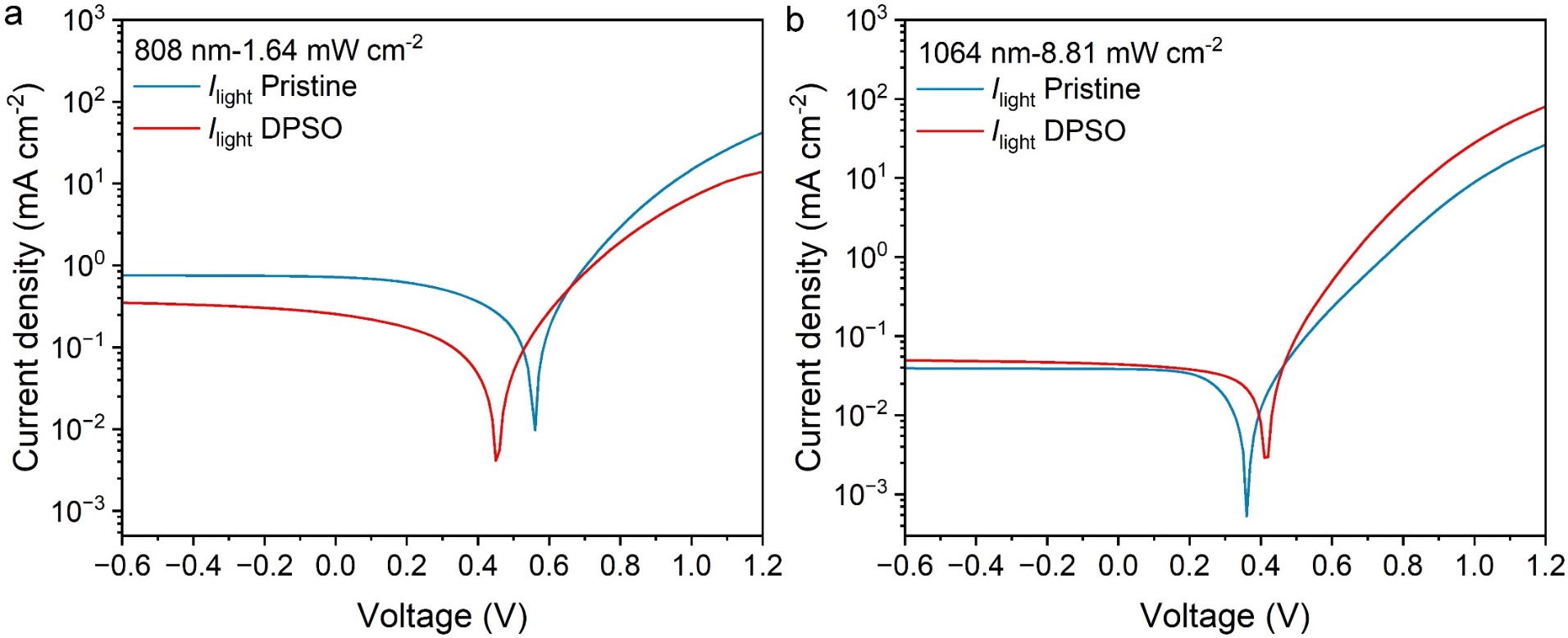


**Fig. S10 Photoresponse of the fabricated NIR photodetectors.** Current density-voltage characteristics of the fabricated photodetector under **a,** 808 nm, and **b,** 1064 nm illumination.

**
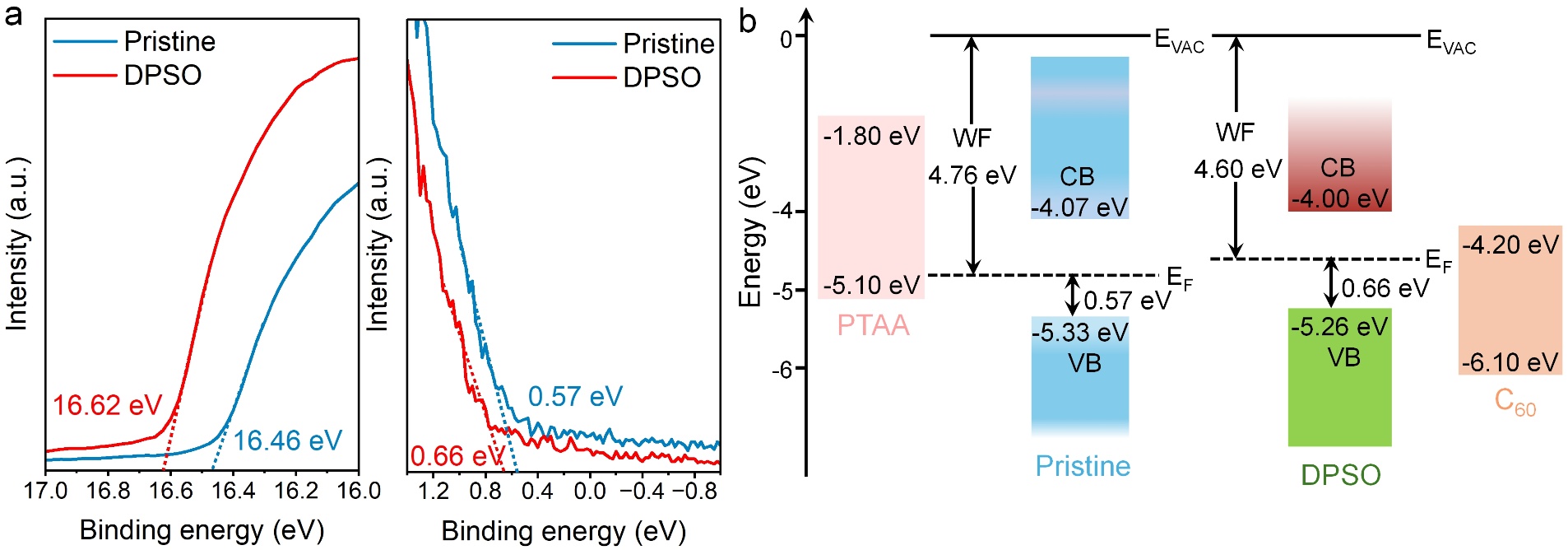
**

**Fig. S11 Energy band alignment of perovskite films. a,** UPS results of perovskite films. **b,** Energy-level diagram of perovskite films along with the adjacent charge transport layers.


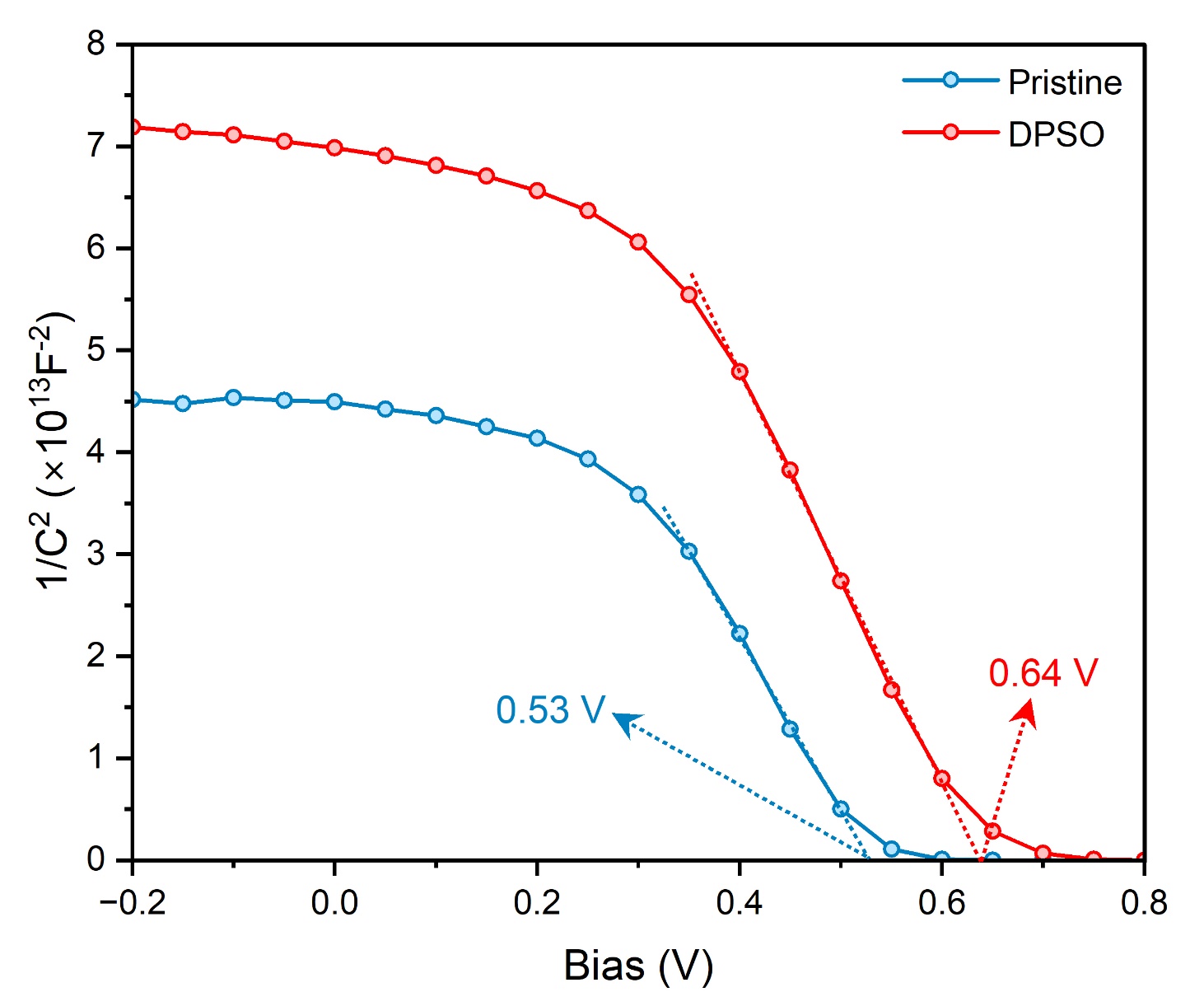


**Fig. S12** Mott-Schottky plots of the pristine and DPSO-modulated photodetector.

**
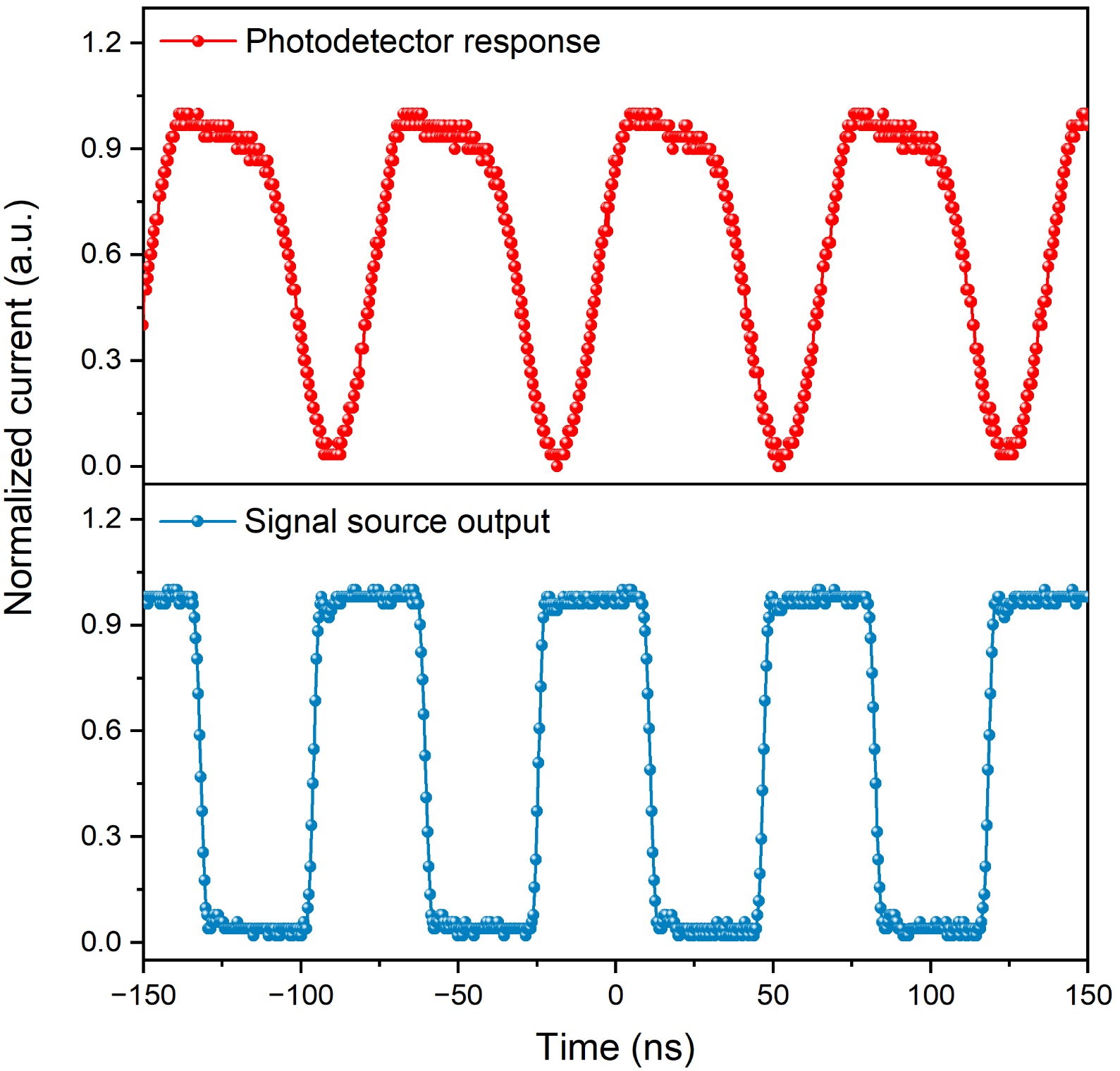
**

**Fig. S13** Temporal response of the fabricated Sn-Pb perovskite photodetector and the output of signal source.

**
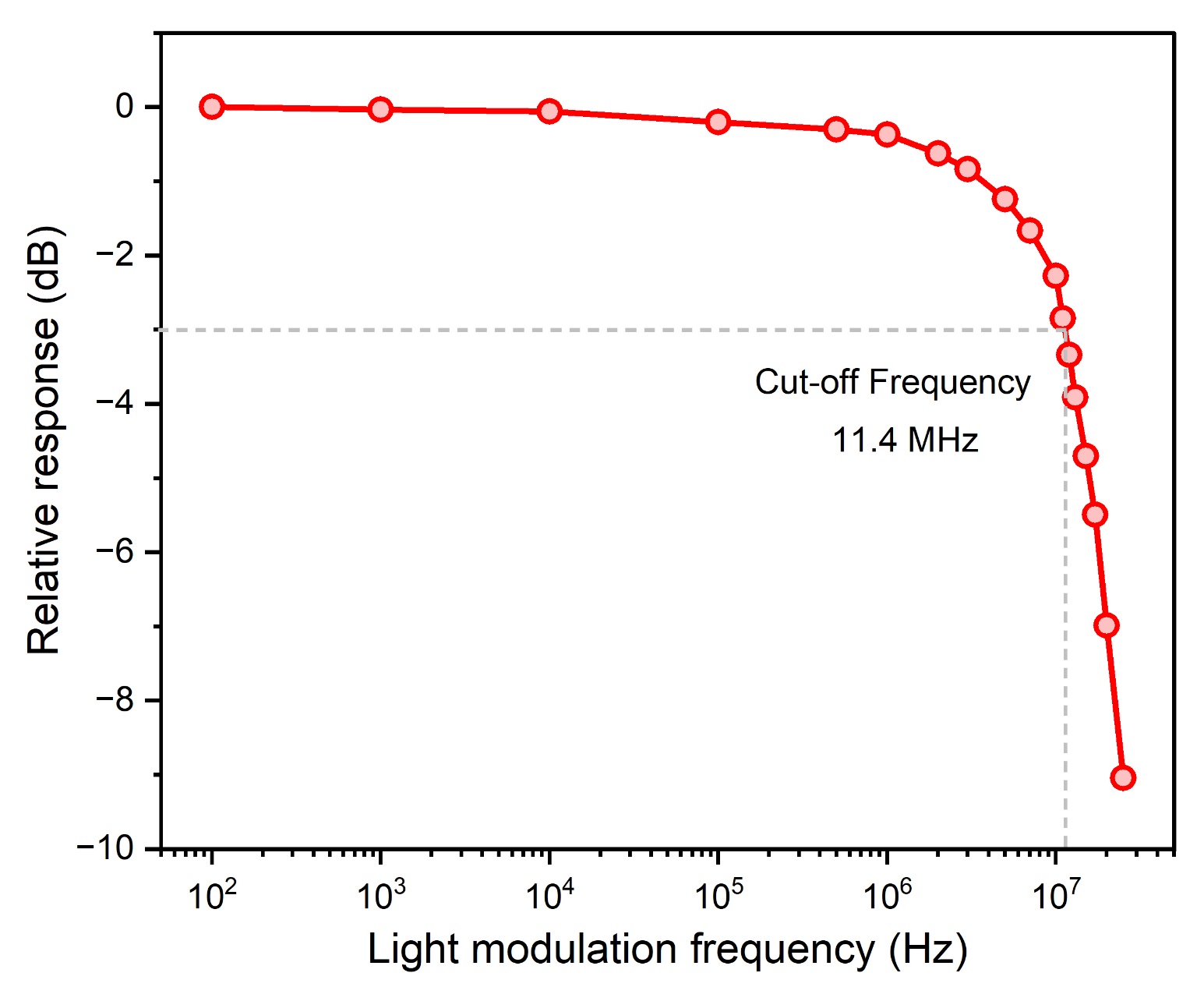
**

**Fig. S14** Response bandwidth of the fabricated Sn-Pb perovskite photodetector.

**
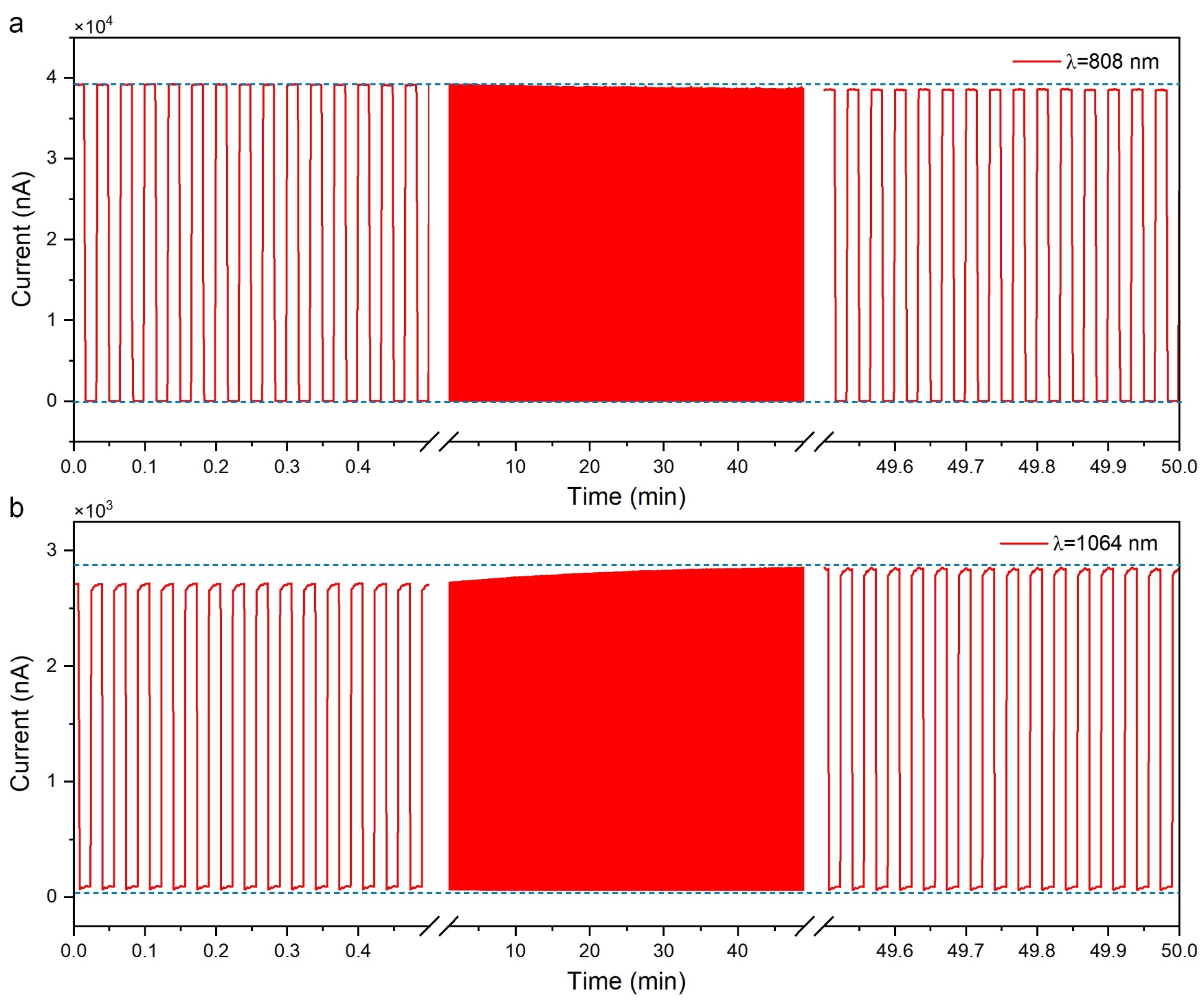
**

**Fig. S15 Continuous on/off response current tracking of the optimal photodetector at the wavelength of a,** 808 nm (1.20 mW cm^−2^) and **b,** 1064 nm (8.33 mW cm^−2^) under zero bias for 50 min.

**
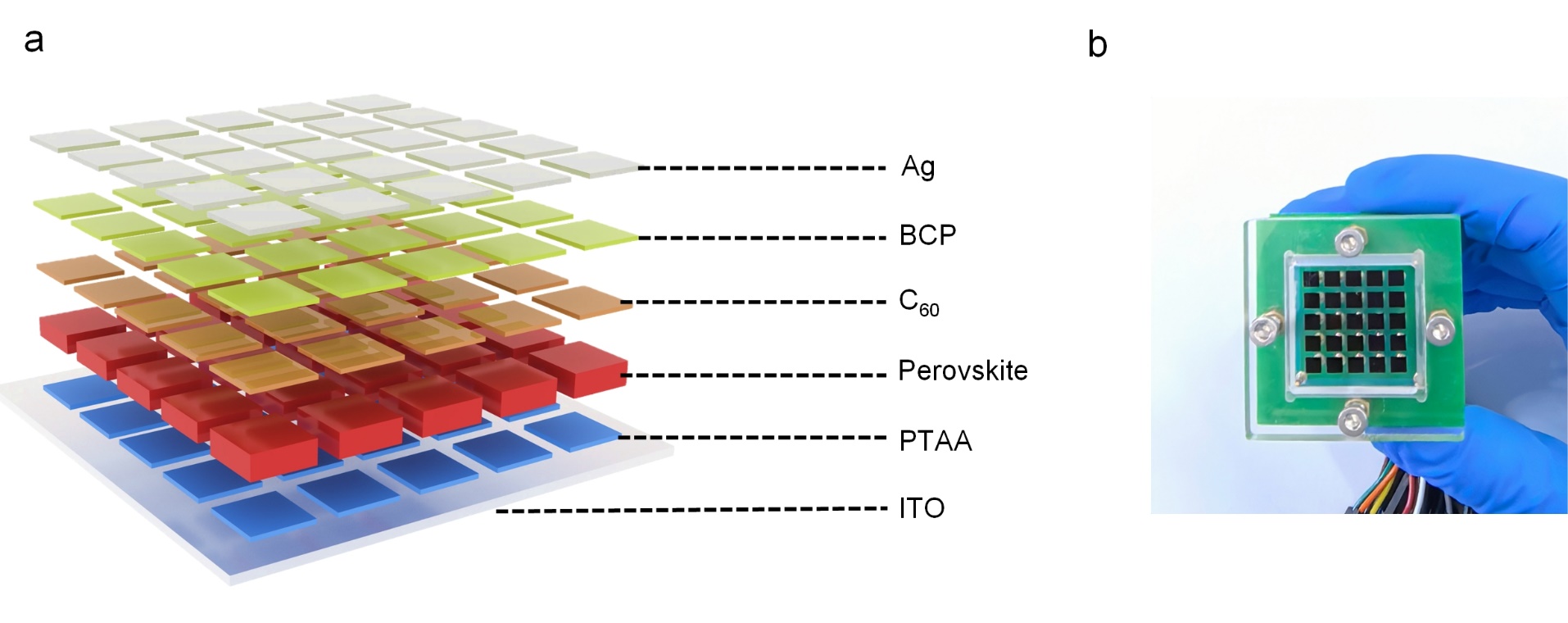
**

**Fig. S16 Structure of the photodetector arrays. a,** The device structure diagram of the photodetector arrays. **b,** Image of the manufactured photodetector arrays.

**
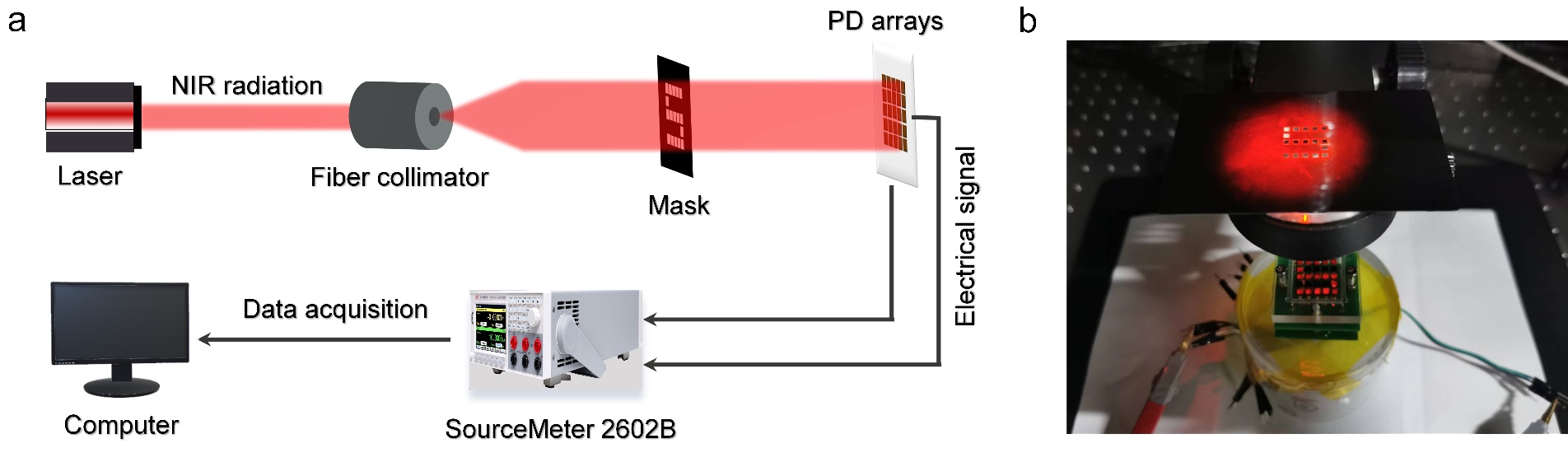
**

**Fig. S17 The setup of imaging system for the fabricated perovskite NIR photodetector (PD) arrays. a**, Schematic diagram of the measurement system. **b**, Photograph of the measurement system.

**
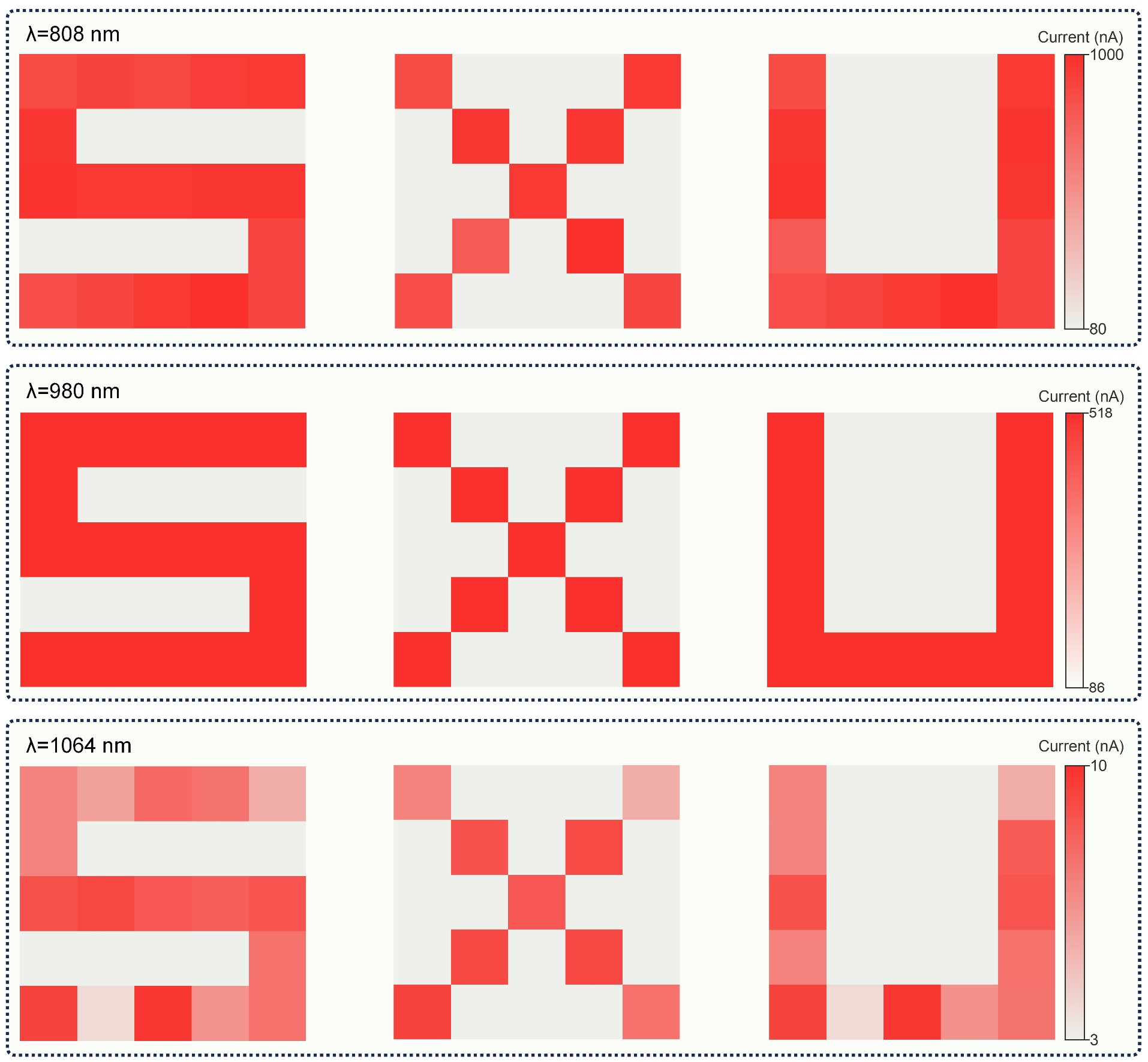
**

**Fig. S18** Imaging of the 5×5 perovskite NIR photodetector arrays for the designed pattern with typical letters of “S”, “X”, and “U”, operated at zero bias under various wavelength.

**
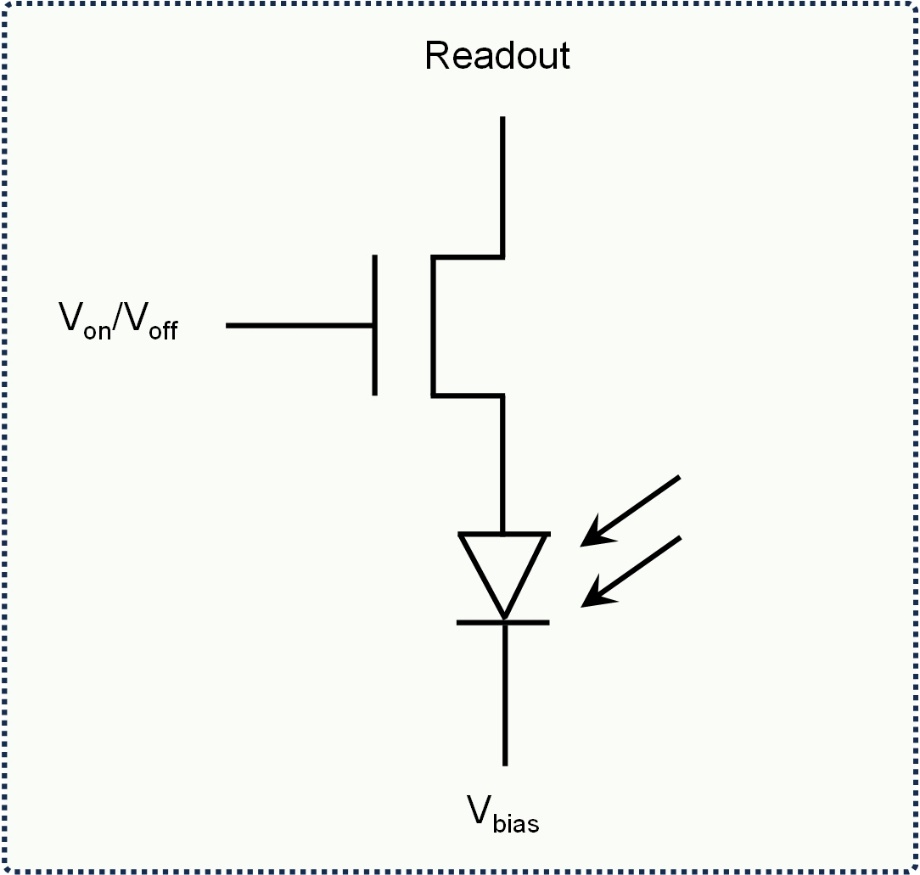
**

**Fig. S19** Circuit diagram of one pixel in the used TFT backplane.

**
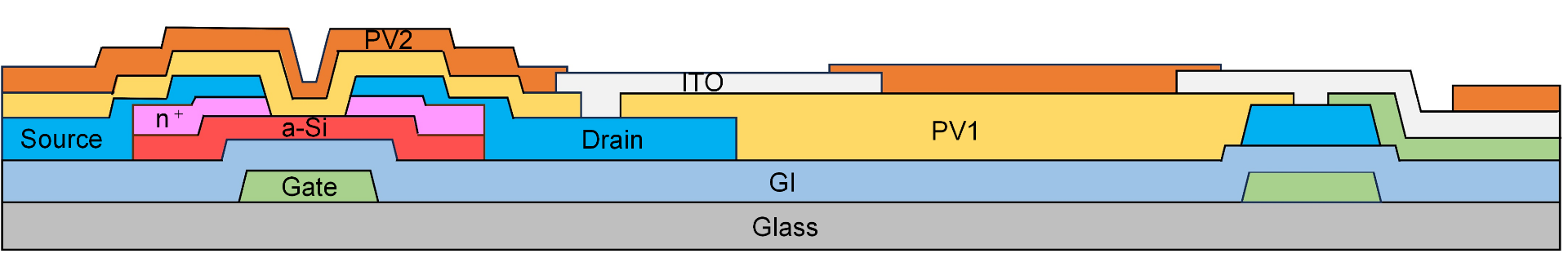
**

**Fig. S20** Cross-sectional diagram of the TFT backplane. PV1, PV2 and GI are SiN_x_ layers for insulating layers.


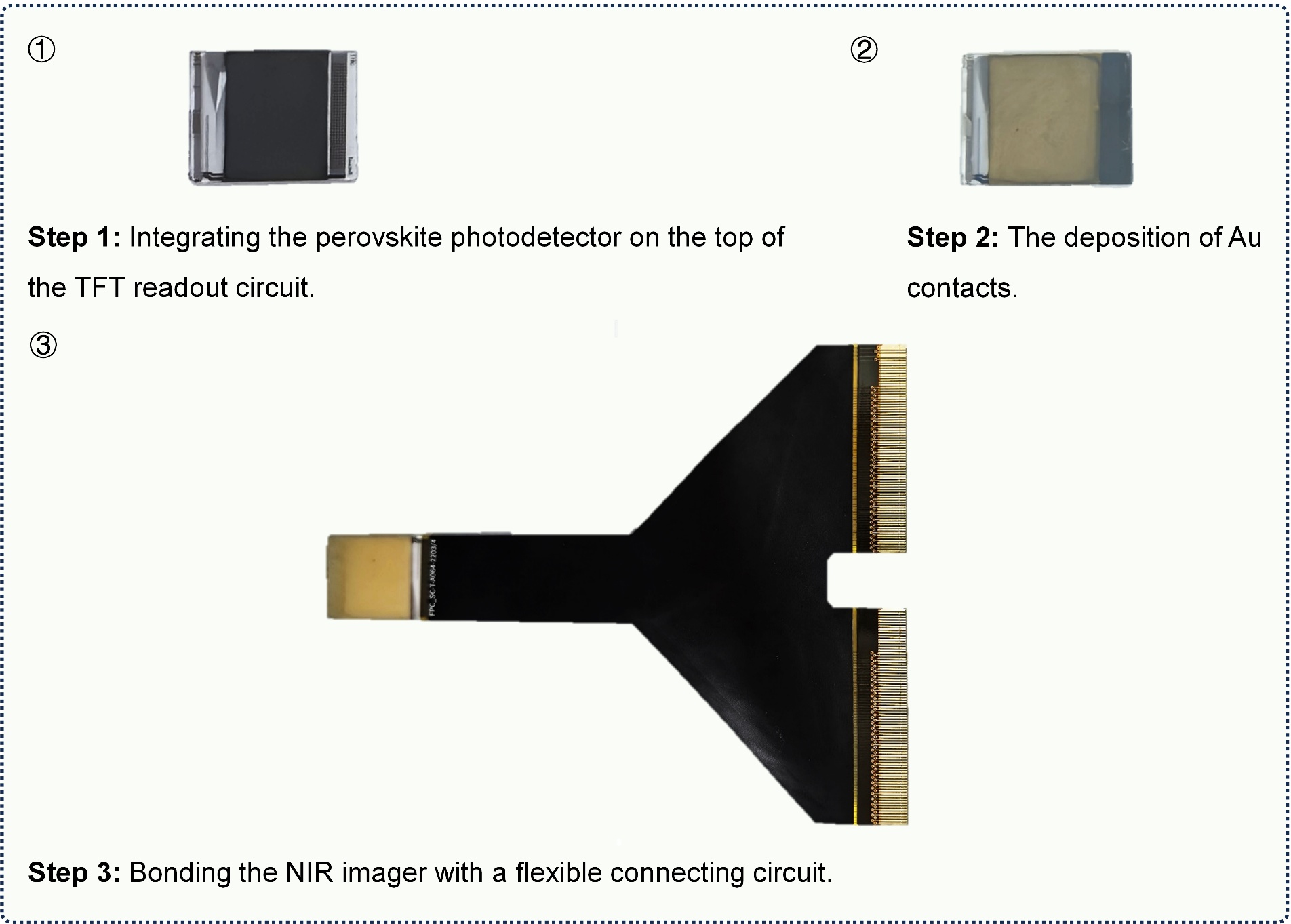


**Fig. S21** The monolithically integration of the perovskite photodetector on the TFT readout circuit and the bonding process.

**
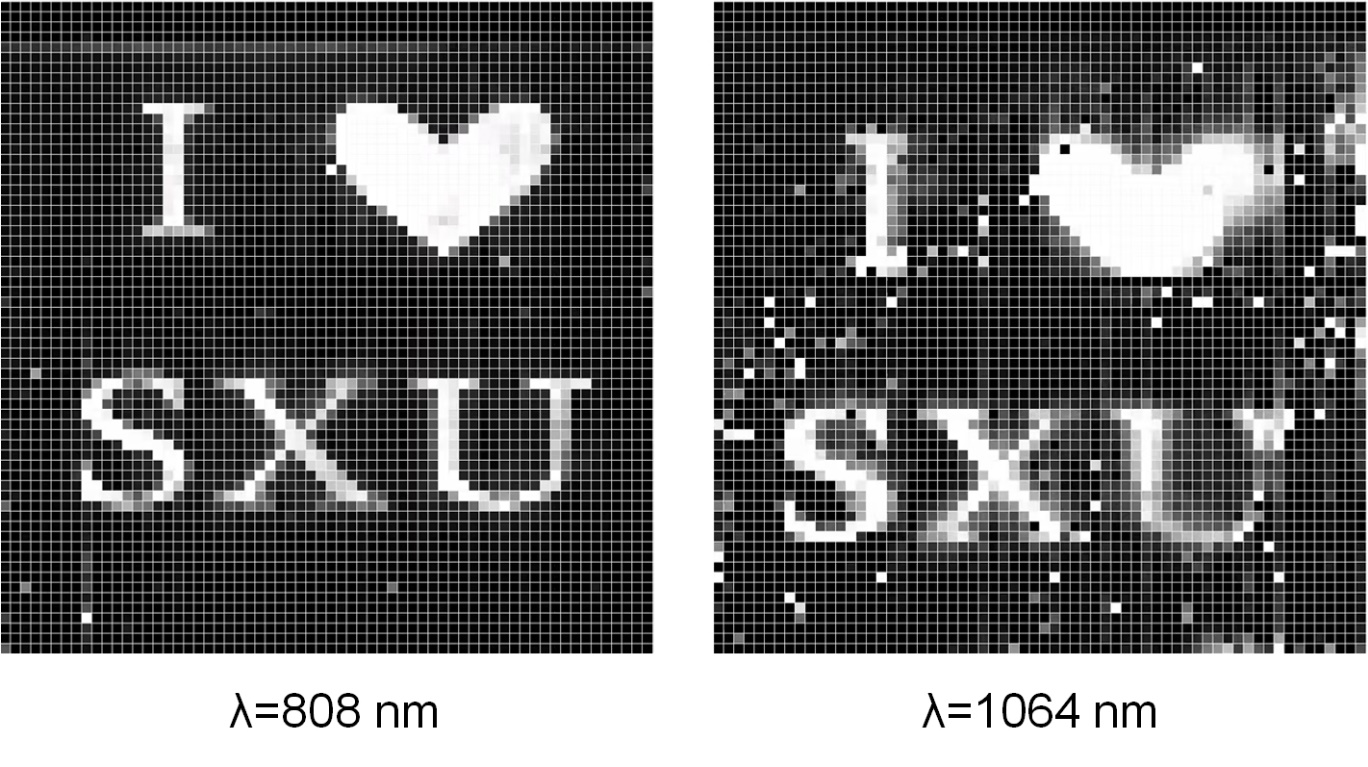
**

**Fig. S22** Image of the imager for a designed pattern of “I ♥ SXU” operated at a wavelength of 808 nm (5.01 mW cm^−2^) and 1064 nm (66.24 mW cm^−2^).

**Table S1.** Summary of TRPL lifetimes for the Pristine and DPSO-modulated perovskite films.

| **Samples** | **A_1_** | $\boldsymbol{\tau}_{\mathbf{1}}$ **(ns)** | **A_2_** | $\boldsymbol{\tau}_{\mathbf{2}}$**(ns)** | $\boldsymbol{\tau}_{\boldsymbol{ave}}$**(ns)** |
| --- | --- | --- | --- | --- | --- |
| Pristine | 0.62 | 44.72 | 0.32 | 150.28 | 111.67 |
| DPSO | 0.35 | 171.23 | 0.59 | 535.95 | 477.84 |

**Table S2.** Summary of TRPL lifetimes for the Pristine and DPSO-modulated perovskite films with different carrier quenching layers.

| **Samples** | **A_1_** | $\boldsymbol{\tau}_{\mathbf{1}}$ **(ns)** | **A_2_** | $\boldsymbol{\tau}_{\mathbf{2}}$**(ns)** | $\boldsymbol{\tau}_{\boldsymbol{ave}}$**(ns)** |
| --- | --- | --- | --- | --- | --- |
| PTAA/Perovskite (pristine) | 0.81 | 4.02 | 0.12 | 31.74 | 18.96 |
| Perovskite (pristine)/C_60_ | 0.64 | 4.56 | 0.33 | 12.47 | 9.19 |
| PTAA/Perovskite (DPSO) | 0.62 | 4.53 | 0.28 | 18.67 | 13.73 |
| Perovskite (DPSO)/C_60_ | 0.74 | 3.27 | 0.17 | 13.86 | 8.49 |

**Table S3.** Trap state density calculation of perovskite films.

| **Hole/electron** | **Samples** | ***V*_TFL_ (V)** | ***N*_trap_ (cm^-3^)** |
| --- | --- | --- | --- |
| Hole | Pristine | 0.367 | 1.29 × 10^16^ |
|  | DPSO | 0.292 | 1.0 × 10^16^ |
| Electron | Pristine | 0.384 | 1.35 × 10^16^ |
|  | DPSO | 0.234 | 8.05 × 10^15^ |

**Table S4.** Performance summary of the commercial and the fabricated Sn-Pb perovskite NIR photodetectors.

| **Functional materials** | **D* (Jones)** | **Response**  **Time (ns)** | **Wavelength (nm)** | **Ref.** |
| --- | --- | --- | --- | --- |
| InGaAs | 6×10^12^ | NA | 700-1700 | [1] |
| Si-FDS1010 | NA | 65 | 350−1100 nm | commercial |
| Ge-FDG03 | 2 × 10^11^ | 600/600 | 800−1800 nm | commercial |
| **FA_0.7_MA_0.3_Sn_0.5_Pb_0.5_I_3_** | **1.20×10^12^** | **14.2/17.1** | **300−1100 nm** | **This work** |

**Note:** 1. The performance of Si-FDS1010 is extracted from:

<https://www.thorlabs.us/thorProduct.cfm?partNumber=FDS1010>

2. The performance of Ge-FDG03 is extracted from:

<https://www.thorlabs.de/thorProduct.cfm?partNumber=FDG03-CAL>

**Table S5.** Summary of the representative performance of the reported perovskite photodetectors.

| **Device structure** | **D* (Jones)** | **Response time (μs)** | **Wavelength (nm)** | **Monolitically integratiuon** | **Ref.** |
| --- | --- | --- | --- | --- | --- |
| ITO/PEDOT:PSS/CH_3_NH_3_PbI_3_/PC_60_BM/C_60_/LiF/Ag | >10^12^ | 1.7/1 | ~800 | No | [2] |
| ITO/PTAA/(FASnI_3_)_0.1_(MAPbI_3_)_0.9_/PCBM/C_60_/BCP/Ag | 7.09×10^10^ | 22.78/20.35 | ~1000 | No | [3] |
| ITO/PEDOT:PSS/FA_0.8_PEA_0.2_SnI_3_/PCBM/BCP/Ag | 2.3×10^11^ | 25/42 | ~900 | No | [4] |
| ITO/Cs_0.05_FA_0.79_MA_0.16_PbI_2_Br/C_60_/SnO_x_/PEDOT:PSS/Cs_0.05_FA_0.79_MA_0.16_PbI_3_/C_60_/BCP/Cu | 6.0×10^11^ | 0.228/0.248 | ~800 | No | [5] |
| rGO/PEDOT:PSS/PEA_2_SnI_4_/ PEDOT:PSS/rGO | 1.92×10^11^ | 6.3×10^5^/3.6×10^6^ | ~750 | No | [6] |
| Au/Cs_2_SnBr_6_ SCs/Au | 2.71×10^10^ | 9.52×10^3^/4.34×10^3^ | ~900 | No | [7] |
| Au/Graphene/MoS_2_/(PEA)_2_SnI_4_/MoS_2_/graphene/Au | 8.09×10^9^ | 3.4×10^4^/3.8×10^4^ | ~800 | No | [8] |
| ITO/PEDOT:PSS/MA_0.5_FA_0.5_Pb_0.5_Sn_0.5_I_3_/PCBM/bis-C_60_/Ag | 10^12^ | 7.4 | 400-940 | No | [9] |
| ITO/CsPbBr_3_ microcrystals/ITO | ~10^11^ | 500/1600 | 400-600 | No | [10] |
| ITO/PTAA/PEAI/Cs_0.05_MA_0.45_FA_0.5_Pb_0.5_Sn_0.5_I_3_/PEAI/C_60_/BCP/Cu | 2.07 × 10^11^ | 0.035 | 300-1050 | No | [11] |
| ITO/PEDOT:PSS/(FASnI_3_)_0.6_(MAPbI_3_)_0.4_/ C_60_/BCP/Ag | 1.1 × 10^12^ | 6.9 | 300-1000 | No | [12] |
| Au/MAPbBr_3_ MWs/Au | 4.1 × 10^11^ | 1.6×10^3^/6.4×10^3^ | ~620 | Yes | [13] |
| ITO/(BA)_2_(MA)Pb_2_I_7_ | 1×10^11^ | 1.25×10^5^/7.4×10^4^ | ~800 | No | [14] |
| Au/CsPbBr_3_ nanowires/PMMA/Au | 1 × 10^11^ | 400/2300 | ~600 | No | [15] |
| Au/TiO_2_/graphene/MAPbI_3_/Au | 4.5 × 10^11^ | 5000 | 260-900 | No | [16] |
| SiO_2_/APTES/MoS_2_/MAPbI_3_/Si | 1.38 × 10^10^ | 6.17×10^6^/4.5×10^6^ | 520-850 | No | [17] |
| **ITO/PTAA/FA_0.7_MA_0.3_Sn_0.5_Pb_0.5_I_3_/C_60_/BCP/Ag** | **1.20 × 10^12^** | **1.4×10^-2^/1.7×10^-2^** | **~1100** | **Yes** | **This work** |

**Note:** Specific detection of the statistical references was evaluated by the noise current rather than the dark current and the response time was tested by the standard square wave.

**References**

1. Saran, R. & Curry, R. J. Lead sulphide nanocrystal photodetector technologies. *Nat. Photon.* **10**, 81-92 (2016).
2. Lin, Q. Q. et al. Low noise, ir‐blind organohalide perovskite photodiodes for visible light detection and imaging. *Adv. Mater.* **12**, 1969-1969 (2015).
3. Chang, Z. Z. et al. Narrow-bandgap Sn-Pb mixed perovskite single crystals for high-performance near-infrared photodetectors. *Nanoscale* **15**, 5053-5062 (2023).
4. He, M. et al. Sn‐based self‐powered ultrafast perovskite photodetectors with highly crystalline order for flexible imaging applications. *Adv. Funct. Mater.* **33**, 2300282 (2023).
5. Moseley, O. D. I. et al. Tunable multiband halide perovskite tandem photodetectors with switchable response. *ACS photonics* **9**, 3958-3966 (2022).
6. Qian, L. et al. A lead-free two-dimensional perovskite for a high-performance flexible photoconductor and a light-stimulated synaptic device. *Nanoscale* **10**, 6837-6843 (2018).
7. Zhou, J. et al. Lead‐free perovskite derivative Cs_2_SnCl_6-x_Br_x_ single crystals for narrowband photodetectors. *Adv. Opt. Mater.* **7**, 1900139 (2019).
8. Fang, C. et al. High-performance photodetectors based on lead-free 2D Ruddlesden-Popper perovskite/MoS_2_ heterostructures. *ACS Appl. Mater. Interfaces* **11**, 8419-8427 (2019).
9. Xu, X. B. et al. High‐performance near‐ir photodetector using low‐bandgap MA_0.5_FA_0.5_Pb_0.5_Sn_0.5_I_3_ perovskite. *Adv. Funct. Mater.* **27**, 1701053 (2017).
10. Yang, B. et al. Ultrasensitive and fast all‐inorganic perovskite‐based photodetector via fast carrier diffusion. *Adv. Mater.* **29**, 1703758 (2017).
11. Zhao, Y. et al. Sensitive and stable tin-lead hybrid perovskite photodetectors enabled by double‐sided surface passivation for infrared upconversion detection. *Small* 2020 **16**, 2001534 (2020).
12. Wang, W. B. et al. Highly sensitive low‐bandgap perovskite photodetectors with response from ultraviolet to the near‐infrared region. *Adv. Funct. Mater.* **27**, 1703953 (2017).
13. Xue, J. et al. Narrowband perovskite photodetector-based image array for potential application in artificial vision. *Nano Lett*. **18**, 7628-7634 (2018).
14. Li, J. Z. et al. Self-trapped state enabled filterless narrowband photodetections in 2D layered perovskite single crystals. *Nat. Commun.* **10**, 806 (2019).
15. Hu, J. B. et al. High-performance self-powered photodetector based on the lateral photovoltaic effect of all-inorganic perovskite CsPbBr_3_ heterojunctions. *ACS Appl. Mater. Interfaces* **15**, 1505-1512 (2022).
16. Li, S. X. et al. Perovskite single‐crystal microwire‐array photodetectors with performance stability beyond 1 year. *Adv. Mater.* **32**, 2001998 (2020).
17. Kang, D. H. et al. An ultrahigh‐performance photodetector based on a perovskite-transition‐metal‐dichalcogenide hybrid structure. *Adv. Mater.* **28**, 7799-7806 (2016).
